# Supplementary material for: Improved Nucleoside (2′-Deoxy)Ribosyltransferases Maximize Enzyme Promiscuity while Maintaining Catalytic Efficiency
Source: ACS Chem Biol. 2025 Oct 17;20(11):2547–53. doi: 10.1021/acschembio.5c00120 (PMC12645435; doi:10.1021/acschembio.5c00120)
Supplement: Supplementary file 1 [file cb5c00120_si_001.pdf]

Supporting information for **"Improved nucleoside (2'-deoxy)ribosyltransferases maximize enzyme promiscuity while maintaining catalytic efficiency"**

Peijun (Gary) Tang[a], Greice M. Zickuhr[b], Alison L. Dickson[b], Christopher J. Harding[c], Suneeta Devi[d], Tomas Lebl[e], David J. Harrison[b,f], Rafael G. da Silva[a] and Clarissa M. Czekster [a,*]

[a]School of Biology, Biomedical Sciences Research Complex, University of St Andrews, St Andrews, Fife KY16 9ST, United Kingdom; [b]School of Medicine, University of St Andrews, North Haugh, St Andrews, KY16 9TF, UK; [c] Institute of Infection, Veterinary and Ecological Sciences, University of Liverpool, L69 3BX, UK; [d] School of Physical Sciences, University of Liverpool, L69 7ZF, UK; [e]School of Chemistry and Biomedical Sciences Research Complex, University of St Andrews and EaStCHEM, North Haugh, St Andrews, Fife, KY16 9ST, United Kingdom; [f]NuCana Plc, Edinburgh, EH12 9DT, United Kingdom

Contents

Materials and methods 4

Abbreviations 4

Materials 4

Additional Methods 4

Cloning, expression and purification of *Ct*NDT mutants 4

Substrate specificity of unusual nucleosides and bases using *Ct*NDT$_{Y7F\text{-}A9S}$ detected by LC-MS. 6

Standard enzyme kinetics of CtNDT with nucleoside substrates using HPLC endpoint assay 7

Estimation of reaction yield by HPLC 8

Determination of K_D for Immucillin-H by differencial scanning fluorimetry 8

Crystallisation, data collection, structure solution, model building, refinement and validation 9

Supporting Tables 10

Table S1. Primers used for mutagenesis 10

Table S2. Masses expected and observed for nucleosides substrates and products. Only reactions that yielded expected products are shown. 12

Table S3. Steady-state parameters for enzyme variants with 2′-deoxyribonucleosides and ribonucleosides. 24

Table S4: Crystallization conditions 25

Table S5: Crystallographic data 26

Supporting Figures 27

Figure S1: Data for reactions to produce nucleosides. Masses indicated in red were assigned to nucleoside products and absent from reactions where bases were omitted. For all graphs “intensity” is in arbitrary ion counts. Left, total ion counts for data acquired in selected ion recording (SIR) mode with m/z set for each expected product with a charge of +1. Right, m/z features contributing to the SIR channel acquired, with the expected m/z shown in red. 27

CtNDT WT 27

CtNDT Y7F – confirmation of activity with ribonucleosides 34

$CtNDT_{Y7F-A9S}$ ... 36

BpNDT ... 43

BpNDT Y5F ... 45

Figure S2: Interactions of *Ct*NDT with transition state analogue DADmeImmH and $CtNDT_{Y7F-A9S}$ with substrate analogue ImmH. ... 46

Figure S3: Ligand binding site of NDT enzymes. a and b, residues interacting with ribosyl moiety of nucleoside susbtrates. ... 48

Figure S4. HPLC and raw kinetic data for all enzyme variants tested. ... 54

Figure S5: Proof of concept for nucleoside production using $CtNDT_{Y7F-A9S}$. ... 55

Figure S6: Reaction tests to improve reaction yield and optimize nucleoside product formation ... 57

Figure S7: SDS-PAGE for all proteins used in this work. ... 58

Figure S8: Intact protein mass spectrometry for all proteins employed in this work. 61

Supporting References ... 62

Materials and methods

Abbreviations

2′-deoxyadenosine (2′-dAdo), 2′-deoxyguanosine (2′-dGuo), 2′-deoxyinosine (2′-dIno), 2´-dUridine (2′-dUrd), 2´-dCytidine (2′-dCyd), 2′-dThymidine (2′-dThd), Adenine (Ade), Guanine (Gua), Hypoxanthine (Hyp), Cytidine (Cyt), Adenosine (Ado), Guanosine (Guo), Inosine (Ino), Uracil (Ura), Uric acid (UricA), liquid-chromatography-mass spectrometry (LC-MS), polyethylene glycol (PEG), Immucillin H (ImmH).

Materials

The synthetic gene was ordered from Integrated DNA Technologies (IDT). The chemicals and reagents were provided by Fluorochem, Merck and Fisher Scientific. Isocytidine was provided by Nucana plc.

Additional Methods

Cloning, expression and purification of *Ct*NDT mutants

The original synthetic gene encoding the *Chroococcidiopsis thermalis* NDT (Uniprot code: K9TVX3) and *Bacillus psychrosaccharolyticus* NDT (Uniprot code: A0A3G5BRZ6) were cloned into a pJ411 (*Ct*NDT) or pJ414 (*Bp*NDT) expression plasmid with a cleavable 6-histidine tag at N-terminus. Single mutants *Ct*NDT$_{A9S}$, *Ct*NDT$_{V85S}$, *Ct*NDT$_{Y7A}$, *Ct*NDT$_{P40A}$, double mutant *Ct*NDT$_{Y7F\text{-}A9S}$ and triple mutants *Ct*NDT$_{Y7F\text{-}A9S\text{-}V85}$ and *Ct*NDT$_{Y7F\text{-}A9S\text{-}P40A}$, as well as *Bp*NDT$_{Y5F}$ were cloned with primers listed on Table S1 (generated using NEBaseChanger - New England Biolabs Inc., Massachusetts, USA). Mutagenesis was carried out using the KLD (Kinase–Ligase–DpnI) enzyme mix (M0554S, New England Biolabs Inc., Massachusetts, USA) to remove the DNA template and ligate the new amplicon, followed by transformation into NEB 5alpha cells. All mutations were confirmed by sequencing (Eurofins).

The expression and purification protocols for the mutants are the same as previously described[1], except *Bp*NDT remained in the histidine tag due to protein instability after tag removal[2]. In summary, the purification protocol was as follows:

The synthetic gene encoding the *Chroococcidiopsis thermalis* NDT (Uniprot code: K9TVX3) was ordered as a Gblock (Integrated DNA Technologies) and cloned into a pJ411 expression plasmid with a cleavable 6-histidine tag at the N-terminus by Gibson assembly. Construct design was carried out using NEBuilder (New England Biolabs), following the cloning protocol suggested to amplify the plasmid pJ411 backbone as well as the GBlock to generate an overlap of 20 bp between sequences. Following cloning, gene sequence was confirmed by sequencing (Eurofins). pJ411::*Ct*NDT and pJ414::*Bp*NDT were transformed into *E.coli* BL21 (DE3) cells for overexpression in LB medium with 50 mg/mL kanamycin or 100 mg/mL ampicillin at 37°C with shaking at 180 rpm shaking until cells reached OD_{600} = 0.8. Protein expression was induced by addition of 0.5 mM of IPTG overnight at 16°C with shaking at 180 rpm. Cells were harvested by centrifugation at 12,000 g for 20 min, resuspended in wash buffer (50 mM MES, 250 mM NaCl, 30 mM imidazole, pH 6.5) and lysed using a cell homogenizer (Constant Systems). Following centrifugation for 30 min at 51,000 *g* at 4°C, the supernatant was filtered with a 0.8 mm filter to remove particulates and loaded onto a 5 mL HisTrap column pre-equilibrated with wash buffer. The column was washed with 10 column volumes of wash buffer, and *Ct*NDT and *Bp*NDT were both eluted with 50 mM MES, 250 mM NaCl, 500 mM Imidazole, pH 6.5. After the first HisTrap column, *Bp*NDT and its mutant *Bp*NDT_{Y5F} were dialysed overnight to remove imidazole, then concentrated and stored at -80 °C.

Fractions containing *Ct*NDT were pooled and dialysed with 2 mg/ml Tobacco Etch Virus (TEV) protease (prepared in house)[3] in buffer (50 mM MES, 250 mM NaCl, pH 6.5) overnight at 4°C. The dialysed mixture was loaded onto a 5 mL HisTrap column and the flow-through fractions were collected and analysed by SDS-PAGE. Fractions containing purified *Ct*NDT were pooled, flash frozen and stored at -80 °C (Figure S1). Single mutants *Ct*NDT_{A9S}, *Ct*NDT_{V85S}, *Ct*NDT_{Y7A}, *Ct*NDT_{P40A}, double mutant *Ct*$NDT_{Y7F\text{-}A9S}$ and triple

mutants CtNDT$_{Y7F-A9S-V85}$ and CtNDT$_{Y7F-A9S-P40A}$ were expressed and purified using the same method. Protein concentration was determined by the extinction coefficient at 280nm calculated using Expasy protparam tool[4].

Fractions containing pure *Ct*NDT and *Bp*NDT mutants (>95%) were pooled, flash-frozen and used in the subsequent experiments. Figure S7 depicts all the intact masses for the proteins purified here.

Substrate specificity of unusual nucleosides and bases using CtNDT$_{Y7F-A9S}$ detected by LC-MS.

A standard sample contained CtNDT$_{Y7F-A9S}$ (1 µM), 1 mM nucleoside substrate and 1 mM base substrate (details in Table S3) in a mixed solution (50 µL total) (30 mM CHES, MES and HEPES, pH 6.5) incubated at 65 °C for 2 hours. The reaction was quenched with 200 µL of MeOH and incubated at –80 °C for 15 minutes. Samples were centrifuged at 24000 x g for 10 minutes, and 200 µL of supernatant was removed and evaporated under a stream of nitrogen gas. Sample were resuspended with 75 µL of LC-MS grade H_2O and 10 µL was injected and analysed by LC-MS.

LC conditions: Nucleosides were analysed on a Waters Acquity HSS T3, 1.8 µm, 2.1mm X 100 mm column. A flow rate of 300 µL min^{-1} at initial conditions 99% H_2 O + 0.1% Formic Acid with 1% ACN + 0.1% Formic Acid until 1 min, followed by a gradient step to 50% H_2 O + 0.1% Formic Acid with 50% ACN + 0.1% Formic Acid to 3.5 mins. A cleaning step was carried out with 1% H_2O + 0.1% Formic Acid with 99% ACN + 0.1% Formic Acid for 5.5 mins and then was re-equilibrated to 99% H_2O + 0.1% Formic Acid with 1% ACN + 0.1% Formic Acid to 7 mins. The column temperature was 40°C throughout the run.

MS conditions: Samples were analysed using a Waters Q-ToF Xevo G2XS, using MS^E for data acquisition. The capillary voltage was set at 2.5 kV in positive ion mode. The source and desolvation gas temperatures of the mass spectrometer were set at 120 °C and 500 °C, respectively. The cone gas flow was set to 50 L/hr, whilst the desolvation gas flow was set at 1000 L/hr. An MS^E scan was performed between 50 – 700 *m/z* where function 1 employed

MS analysis whilst function 2 applied a collision energy ramp from 15 to 30 V to perform MS/MS fragmentation. A lock spray signal was measured, and a mass correction was automatically applied by collecting every 10s, averaging 3 scans of 1s each using Leucine Enkephalin as a standard (556.2771 *m/z*).

Standard enzyme kinetics of CtNDT with nucleoside substrates using HPLC endpoint assay

Deoxynucleosides assay: A standard assay contained CtNDT (10 nM) or CtNDT$_{Y7F}$ (50 nM), 10 µM-1000 µM dGuo and a nucleobase (10 mM Ade) in a mixed solution (50 µL total) (30 mM CHES, MES and HEPES, pH 6.5) incubated at 45 °C for 5, 10 and 15 minutes. Michaelis-Menten curves for UricA contained 2 µM CtNDT, 1000 µM 2′d-Ado, UricA (varying as follows: 10, 25, 50, 100, 250, 500, 1000, 2500 µM) in 30 mM CHES, MES and HEPES, pH 6.5.

Ribonucleosides assay: A standard assay contained enzyme [different concentrations of each variant used as follows: CtNDT (1 µM), CtNDT$_{Y7F}$ (0.5 µM for RGua, 1 µM for RIno, 0.8 µM for RAdo), CtNDT$_{Y7A}$ (0.8 µM), *Ct*NDT$_{Y7F\text{-}A9S}$ (1 µM), triple mutants *Ct*NDT$_{Y7F\text{-}A9S\text{-}V85S}$ and *Ct*NDT$_{Y7F\text{-}A9S\text{-}P40A}$ (both at 1 µM), CtNDT$_{P40A}$ (1 µM), CtNDT$_{D62N}$ (1 µM)], varying 10 µM-1000 µM ribonucleoside (RAdo, RGua or RIno) and a base (10 mM Hyp or Ade) in a mixed solution (50 µL total) (30 mM CHES, MES and HEPES, pH 7). Reactions were incubated at 65 °C for 5, 10 and 15 minutes. At different time points, the reaction mixture was quenched with 200 µL of 10 M Urea and centrifuged at 24000 x g for 10 minutes. 100 µL of the mixture was placed into a 96-well round bottom microplate (Agilent Technologies) and 10 µL of each sample was injected into the column in HPLC (Shimadzu) or UHPLC (Thermo Fisher Scientific). All the experiments were done in duplicate. The data were analysed with Chromeleon ver 6.80 or LabSolution ver 5.11 and were fitted and plotted as Michaelis-Menten Equation using Prism 9.0.

HPLC conditions: A HSS T3, 2.5 µm, 50 mm X 4.6 mm column (Waters^{TM}) was used with buffer A (10 mM trimethyl ammonium acetate, pH 7) and buffer B (ACN + 0.1% TFA). A gradient elution was used to separate the compounds: at 0-10 mins, 99% to 85% buffer A and 1% to 15% buffer B, and 10-15 mins,

85% to 0% buffer A and 15% to 100% buffer B. The oven temperature was set at 40 °C and the absorbance was set at 260 nm. The column was equilibrated with buffer A : buffer B (1:99) for at least 15 mins before each injection. Retention times for the reference natural compounds: 2'- dAdo: 8.2 min; 2'-dGuo: 3.8 min; 2'- dIno: 2.5 min; RAdo: 8.1 min; RGuo: 2.5 min; RIno: 2.8 min; Ade: 2.4 min; Gua: 1.4 min; Hyp: 1.4 min.

Estimation of reaction yield by HPLC

Compound identities were confirmed by comparison with commercial standards for both the nucleoside products and the starting materials. Quantification was performed using calibration curves generated with authentic standards. Chromatographic analysis was conducted as previously described, with detection at 260 nm. Peak areas for reactants and products were integrated, and UV absorbance values were converted to molar quantities using the appropriate calibration curves for each standard.

Determination of K_D for Immucillin-H by differencial scanning fluorimetry

Assays were prepared in a MicroAmp™ 96-well optical reaction plate (Fisher Scientific) with a final volume of 20 µL per well, containing buffer (30 mM MES, 250 mM NaCl, pH 6.5) and 5X SYPRO Orange dye (Invitrogen). The concentration of Immucillin-H was varied in a serial dilution, the test plate was sealed with adhesive film and centrifuged for 5 minutes at 500 rpm. Fluorescence emission was recorded using an Applied Biosystems® QuantStudio 1 Real-Time PCR system (λex = 520 nm, λem = 558 nm). Temperature increases were performed over the range of 25–95 °C, with increments of 1 °C/min, individual metling curves were analysed using Applied Biosystems Protein Thermal Shift Software v.1.4 (Thermo Fisher Scientific) to determine individual T_M values. The K_D was determined in GraphPad Prism by using the following equation[5].

$$Y = Bottom + (Top - Bottom)(1 - \frac{P - K_D - X + \sqrt{(P + X + K_D)^2 - 4PX}}{2P})$$

(P: protein concentration; K_D: dissociation constant; Top: maximum temperature; Bottom: minimum temperature; X: ligand concentration)

Crystallisation, data collection, structure solution, model building, refinement and validation

CtNDT$_{Y7F}$ with Immucillin-H – The protein concentration was 10 mg/mL, and ligand Immucillin-H was used at a final concentration of 1 mM. Co-crystals were grown at 20 °C using the sitting drop vapour diffusion technique, with a drop size of 0.3 µl in a 1:1, 1:2 or 2:1 reservoir: protein solution. Crystallisation conditions were from the commercial screen PACT (well D12): 20% w/v PEG 6000; 0.1 M Tris pH 8.0; 0.002 M zinc chloride. Crystals were cryoprotected in mother liquor supplemented with 20% (v/v) ethylene glycol before being flash cooled in liquid nitrogen. Diffraction data were collected at the Diamond Light Source in Oxford, UK, on I04 beamlines. Data reduction and processing were completed using the xia2 suite.

CtNDT$_{Y7F_A9S}$ with cordycepin – The protein concentration was 10 mg/mL, and ligand cordycepin was used at a final concentration of 1 mM. Co-crystals were grown at 20 °C using the sitting drop vapour diffusion technique, with a drop size of 0.3 µl in a 1:1, 1:2 or 2:1 reservoir: protein solution. Crystallisation conditions were 21.429% PEG Smear, 0.1 M Bis-Tris Propane pH 8.33. Crystals were cryoprotected in mother liquor supplemented with 20% (v/v) ethylene glycol before being flash cooled in liquid nitrogen. Diffraction data were collected at the Diamond Light Source in Oxford, UK, on I04 beamline. Data reduction and processing were completed using the xia2 suite. The structure was solved by molecular replacement with PHASER searching for four monomers in the ASU. The search component was CtNDT (pdb 8PQS), which had been modified sculptor and manually truncated in COOT.

For both, structures were solved by molecular replacement with PHASER searching for four monomers in the ASU. The search component was CtNDT (pdb 8PQS), which had been modified sculptor and manually truncated in COOT. Protein structures were built/ modified using COOT, with cycles of refinement in PHENIX. Restraints for the ligand were generated using

ACEDRG, available at the CCP4 cloud server. Crystallographic data are shown in Table S4.

Supporting Tables

Table S1. Primers used for mutagenesis

Name	5'-3' Primer Sequence
$CtNDT_{Y7F}$ Forward	TATTTTCCTTGCCAGCCCTTATGGTTTTAG
$CtNDT_{Y7F}$ Reverse	CAAGGAAAATAATCTTACGTTTCATCCCCTGG
$CtNDT_{Y7A}$ Forward	TAAGATTATTGCCCTTGCCAGCC
$CtNDT_{Y7A}$ Reverse	CGTTTCATCATTCCTGTAAG
$CtNDT_{P40A}$ Forward	GGTTTGGGAAGCATTTGCTCGTAATAAC
$CtNDT_{P40A}$ Reverse	TCGATCCCCAGTGCCTCT
$CtNDT_{Y7F\text{-}A9S}$ Forward	TATTTTCCTTAGCAGCCCTTATGG
$CtNDT_{Y7F\text{-}A9S}$ Reverse	ATCTTACGTTTCATCATTCC
$CtNDT_{Y7F\text{-}A9S\text{-}V85S}$ Forward	AGATGAGGGCAGTATGGTAGAATTGGGTATGGC
$CtNDT_{Y7F\text{-}A9S\text{-}V85S}$ Reverse	GGGGGCGTTCCATTAACC
BpNDT NDT Forward	GAAATAATTTTGTTTAACTTTTGGAATTCCATATG CACCAC
BpNDT NDT Reverse	CAAGGGGTTATGCTAGGGGGCCCCAACTTTCA CTTCAC
pJ414 NDT Forward	CCCCCTAGCATAACCCCTTG
pJ414 NDT Reverse	AAAAGTTAAACAAAATTATTTCTAGAGGGGAATTG
$BpNDT_{Y5F}$ Forward	GGCAAAAATCTTTTTGGCGTCAC
$BpNDT_{Y5F}$ Reverse	ATTCACTTCACAGCTTTAAG

Table S2. Masses expected and observed for nucleosides substrates and products. Only reactions that yielded expected products are shown.

Product expected	Nucleoside donor	Nucleobase	Theoretical Mass Nucleoside product [M+H]/[M-H]	Experimental Mass Nucleoside product [M+H]/[M-H]	ppm deviation	Nucleoside product
**WT-*Ct*NDT**						
2’-deoxy-2		Purine	237.0982	237.0980	-0.843	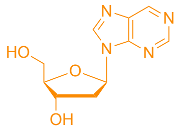
2’-deoxy-3	2′-deoxy adenosine	2-fluoroadenine	270.0998	270.100	0.740	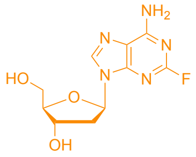
2’-deoxy-4		6-amino-7H-purin-2-ol (isoguanine)	268.1051	268.105	-0.373	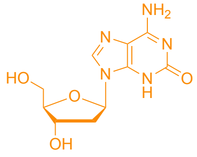

2’-deoxy-5		1-methyl-3,7-dihydro-purine-2,6-dione	283.1038	283.104	0.706	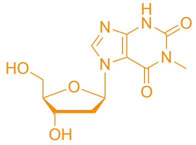
2’-deoxy-6		N-(7H-Purin-6-yl)benzamide	356.1353	356.135	-0.842	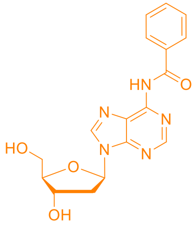
2’-deoxy-7		N, N-dimethyl-7H-purine-6-amine	280.1408	280.141	0.714	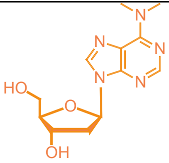
2’-deoxy-8		5-Nitrobarbituric acid	290.0618	290.062	0.690	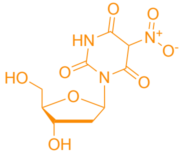
2’-deoxy-9		6-(benzyloxy)-9H-purine	343.1408	343.140	-2.331	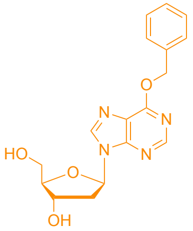

2’-deoxy-11		4,5,6,7-tetrahydro-1H-pyrazolo[4,3,-c]pyridine	240.1348	240.1349	0.416	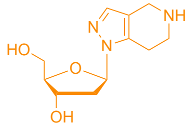
2’-deoxy-12	2′-deoxyguanosine	2,5,6,7,8,9-hexahydro-3H-cyclohepta[c]pyridazine-3-one	281.1501	281.150	-0.356	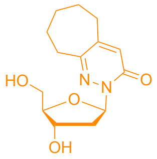
2’-deoxy-13		1,4,5,6-Tetrahydropyrrolo[3,4-c]pyrazole	226.1191	226.120	3.980	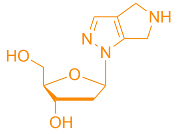
2’-deoxy-14		5-chlorobenzotriazole	270.0639	270.064	0.111	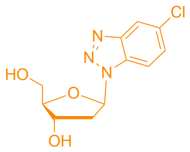
2’-deoxy-15	2′-deoxy adenosine	6-mercaptopurine	269.0708	269.070	-2.973	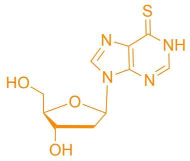
2’-deoxy-16		uric acid*	283.0679	283.068	0.353	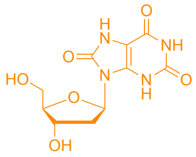

2’-deoxy-19		6-chloro-3-methyluracil*	275.0435	275.043	-1.817	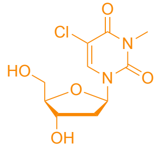
2’-deoxy-21	2′-deoxyuridine	5-azacytosine	229.0938	229.094	0.873	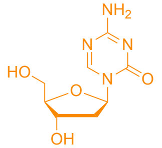
2’-deoxy-22	2′-deoxy adenosine	3-aminopyridin-2(1H)-one	227.1026	227.102	-2.677	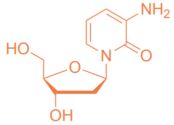
2’-deoxy-38		IBMX	339.1668	339.167	0.589	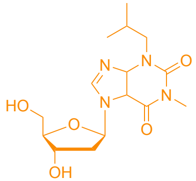
2’-deoxy-47		4-hydroxy-7-azaindole	251.1032	251.1038	2.389	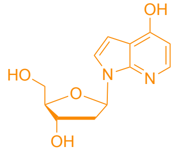
2’-deoxy-48		5-chloro-7H-pyrrolo[2,3-d]pyrimidine	270.0645	270.0657	4.443	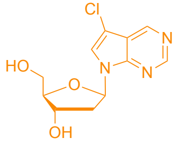

2’-deoxy-54		2,6-diaminopurine	267.1208	267.1214	2.246	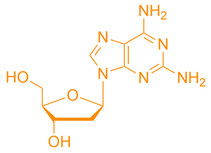
Adenosine	Guanosine	adenine	268.1040	268.105	3.711	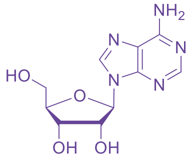
Adenosine	Inosine	adenine	268.1040	268.105	3.711	
Cytidine	Adenosine	cytosine	244.0927	244.093	0.934	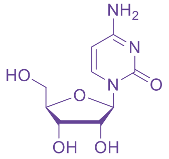
2'-amino-2’-deoxy-adenosine	2′-amino-2’-deoxyuridine	adenine	267.1205	267.120	-1.872	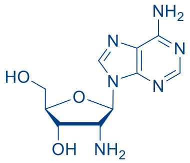
3′-deoxyguanosine	3′-deoxyadenosine (Cordycepin)	guanine	268.104005	268.104	-0.018	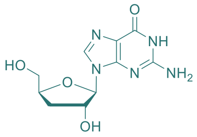
**CtNDT$_{Y7F}$**						

Cytidine	Adenosine	cytosine	244.0927	244.093	0.934	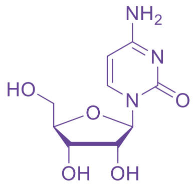
Adenosine	Guanosine	adenine	268.1040	268.105	3.711	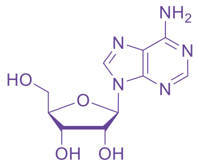
Adenosine	Inosine	adenine	268.1040	268.105	3.711	
**CtNDT$_{Y7F-A9S}$**						
3'-deoxy-2	3′-deoxyadenosine (Cordycepin)	purine	237.0981	237.098	-0.422	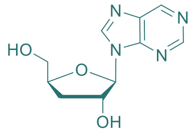
3'-deoxy-3		2-fluoroadenine	270.0998	270.100	0.740	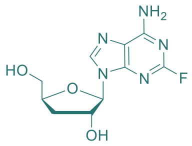
3'-deoxy-5		1-methyl-3,7-dihydro-purine-2,6-dione	283.1038	283.104	0.706	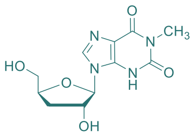

3’-deoxy-7		N, N-dimethyl-7H-purine-6-amine	280.1408	280.141	0.714	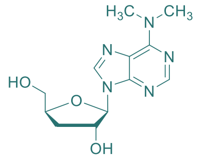
3’-deoxy-9		6-(benzyloxy)-9H-purine	343.1408	343.141	0.582	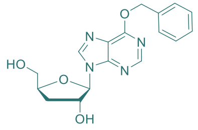
3’-deoxy-10		1H-Indazole	235.1077	235.108	1.301	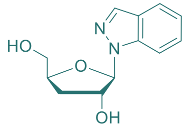
3’-deoxy-15		6-mercaptopurine	269.0708	269.070	-2.973	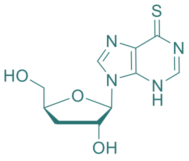
3’-deoxy-38		IBMX	339.1668	339.166	-2.359	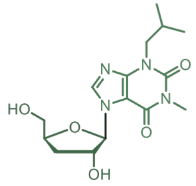
3’-deoxy-54		2,6-diaminopurine	267.1208	267.1211	1.123	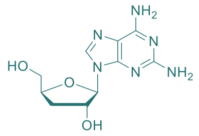

Ribo-1	Adenosine	2,6-dichloropurine	321.0158	321.015	-2.492	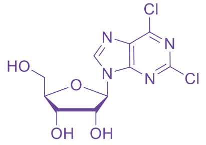
Ribo-2		purine	253.0938	253.093	-3.161	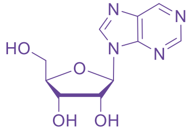
Ribo-3		2-fluoroadenine	286.0948	286.095	0.699	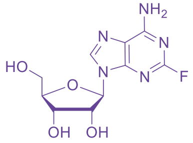
Ribo-5		1-methyl-3,7-dihydro-purine-2,6-dione	299.0988	299.099	0.669	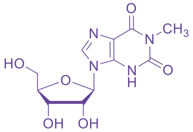
Ribo-6		N-(7H-Purin-6-yl)benzamide	372.1308	372.1322	3.762	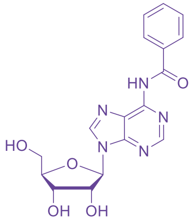
Ribo-7		N, N-dimethyl-7H-purine-6-amine	296.1358	296.136	0.675	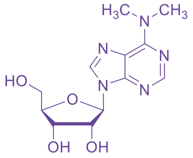

Ribo-9		6-(benzyloxy)-9H-purine	359.1358	359.135	-2.228	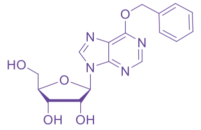
Ribo-10		1H-Indazole	251.1028	251.103	0.796	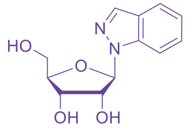
Ribo-13		1,4,5,6-tetrahydropyrrolo[3,4,-c] pyrazole	242.1138	242.1148	4.130	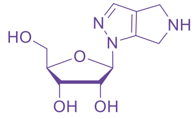
Ribo-14		5-chlorobenzotriazole	286.0588	286.058	-2.796	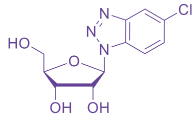
Ribo-15		6-mercaptopurine	285.0658	285.065	-2.806	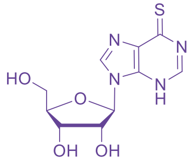
Ribo-38		IBMX	355.1618	355.162	0.563	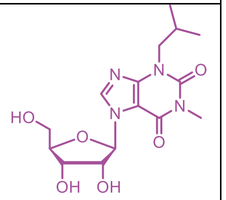

ribo-47		4-hydroxy-7-azaindole	267.0978	267.0969	-3.369	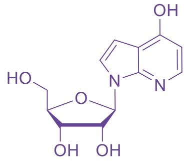
ribo-54		2,6-diaminopurine	283.1158	283.1163	1.766	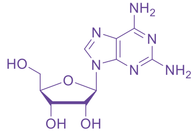
	**WT-*Bp*NDT**					
2’-deoxy-18	2′-deoxyguanosine	5-fluorouracil*	245.0572	245.057	-0.816	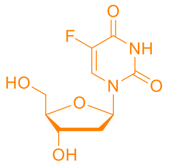
2’-deoxy-20		isocytosine	228.0988	228.098	-3.507	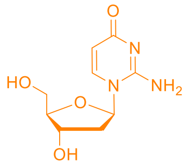
2’-deoxy-21		5-azacytosine	229.0938	229.094	0.873	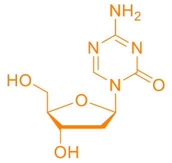

2’-deoxy-23		2-thiouracil*	243.0442	243.044	-0.823	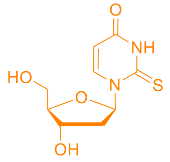
2’-deoxy-24		6-amino-3-methyluracil*	256.0933	256.094	2.733	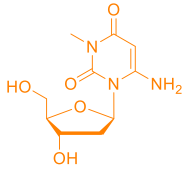
2’-deoxy-26		pyrimidin-4(3H)-one	213.0878	213.087	-3.754	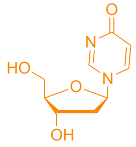
2’-deoxy-29		5-chlorouracil*	261.0282	261.027	-4.597	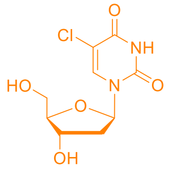
2’-deoxy-30		5-iodouracil*	352.9632	352.963	-0.566	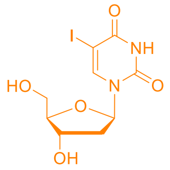

2’-deoxy-32		5-bromouracil*	306.9928	306.992	-2.606	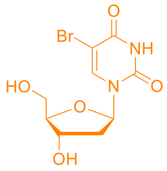
2’-deoxy-43		5-(2-chloroethyl)-1H-tetrazole*	247.0592	247.059	-0.810	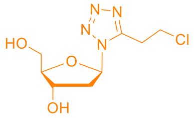
	**BpNDT Y5A**					
Ribo-18	Guanosine	5-fluorouracil	261.0522	261.052	-0.766	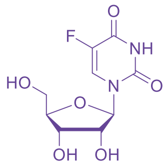

*negative mode

Table S3. Steady-state parameters for enzyme variants with 2′-deoxyribonucleosides and ribonucleosides.

Concentrations of each variant used as follows: CtNDT (1 µM), $CtNDT_{Y7F}$ (0.5 µM for RGua, 1 µM for RIno, 0.8 µM for RAdo), $CtNDT_{Y7A}$ (0.8 µM), *Ct*$NDT_{Y7F\text{-}A9S}$ (1 µM), triple mutants *Ct*$NDT_{Y7F\text{-}A9S\text{-}V85S}$ and *Ct*$NDT_{Y7F\text{-}A9S\text{-}P40A}$ (both at 1 µM), $CtNDT_{P40A}$ (1 µM), $CtNDT_{D62N}$ (1 µM).

Enzyme	Donor	Acceptor	K_M (mM)	k_{cat} (s^{-1})	k_{cat}/K_M ($mM^{-1}s^{-1}$)
CtNDT	2′-dGuo	Ade	0.69 ± 0.14	6.91 ± 0.72	10.01 ± 2.28
	RAdo	Hyp	0.25 ± 0.05	0.03 ± 0.02	0.12 ± 0.09
	RGua	Ade	1.09 ± 0.31	0.06 ± 0.01	0.06 ± 0.02
	RIno		0.92 ± 0.24	0.01 ± 0.01	0.01 ± 0.01
	2′-dAdo	Uric acid	1.46 ± 0.21	0.32 ± 0.02	0.22 ± 0.03
$CtNDT_{Y7F}$	2′-dGuo	Ade	0.67 ± 0.08	0.21 ± 0.01	0.32 ± 0.40
	RAdo	Hyp	0.11 ± 0.01	0.04 ± 0.01	0.33 ± 0.03
	RGua	Ade	0.10 ± 0.02	0.02 ± 0.01	0.20 ± 0.04
	RIno		0.71 ± 0.17	0.03 ± 0.01	0.04 ± 0.01
$CtNDT_{Y7A}$	RIno	Ade	0.53 ± 0.01	0.02 ± 0.01	0.04 ± 0.01
Ct$NDT_{Y7F\text{-}A9S}$			0.62 ± 0.10	0.05 ± 0.01	0.08 ± 0.04
Ct$NDT_{Y7F\text{-}A9S\text{-}V85S}$			0.84 ± 0.21	0.04 ± 0.01	0.05 ± 0.02
Ct$NDT_{Y7F\text{-}A9S\text{-}P40A}$			0.06 ± 0.01	0.004 ± 0.001	0.07 ± 0.01
$CtNDT_{P40A}$			0.10 ± 0.02	0.01 ± 0.01	0.07 ± 0.02
$CtNDT_{D62N}$			0.80 ± 0.09	0.002 ± 0.001	0.003 ± 0.001

Table S4: Crystallization conditions

PDB accession code	Protein	Protein concentraiton	**Ratio protein/precipitant**	**Crystallization condition**	**Ligand and concentration for co-crystallization**
9EMW	Y7F	10 mg/ml	1:1	0.1 M Tri, pH 8.0, 20% PEG 6000, 0.002 M zinc chloride	1 mM ImmH, co-crystals
9EMX	DM (Y7F A9S)	10 mg/ml	1:1	0.1 M Bis-Tris Propane, pH 8.33, 21.429% PEG Smear High, 0.05 M magnesium chloride	1 mM Cordycepin, co-crystals

Table S5: Crystallographic data

Property	Value	Value
**pdb code**	9EMX - *Ct*NDT$_{Y7F-A9S}$ bound to Cordycepin	9EMW - CtNDTY7F bound to ImmH
Space group	P 32	P 63
Cell constants	97.38Å 97.38Å 66.46Å	135.76Å 135.76Å 87.55Å
a, b, c, *α*, *β*, *γ*	90.00◦ 90.00◦ 120.00◦	90.00◦ 90.00◦ 120.00◦
Resolution (Å)	42.17 – 1.77	48.8 = 2.51
	84.33 – 1.77	70.22 - 2.51
% Data completeness	94.0 (42.17-1.77)	99.8 (48.80-2.51)
(in resolution range)	99.0 (84.33-1.77)	99.8 (70.22-2.51)
R*merge*	0.18	0.16
< I/σ(I) > 1	0.96 (at 1.76Å)	1.23 (at 2.51Å)
Refinement program	PHENIX 1.19.2-4158	PHENIX (1.21.2-5419)
R, R*free*	0.188 , 0.211	0.212, 0.262
	0.187 , 0.210	0.213, 0.26
R_{free} test set	2064 reflections (3.01%)	1566 reflections (4.98%)
CC1/2	1	1
Wilson B-factor (Å2)	34.9	61.4
Anisotropy	0.382	0.262
Bulk solvent k_{sol}(e/Å3), B_{sol}(Å2)	0.31 , 40.2	0.31 , 37.1
L-test for twinning2	$< \|L\| >$ = 0.49, $< L^2 >$ = 0.33	$< \|L\| >$ = 0.51, $< L^2 >$ = 0.34
Estimated twinning fraction	0.014 for -h,-k,l	0.035 for h,-h-k,-l
	0.033 for h,-h-k,-l	
	0.018 for -k,-h,-l	
F_o,F_c correlation	0.97	0.93
Total number of atoms	5540	5227
All atom clashscore	8.4	5.4
Average B, all atoms (Å2)	41	66
Clashscore all atoms	5.39	4,76
Molprobity score	1.29	1.57

Supporting Figures

Figure S1: Data for reactions to produce nucleosides. Masses indicated in red were assigned to nucleoside products and absent from reactions where bases were omitted. For all graphs "intensity" is in arbitrary ion counts. Left, total ion counts for data acquired in selected ion recording (SIR) mode with m/z set for each expected product with a charge of +1. Right, m/z features contributing to the SIR channel acquired, with the expected m/z shown in red.

CtNDT WT

Product 2'-deoxy-2

2'dAdo + purine

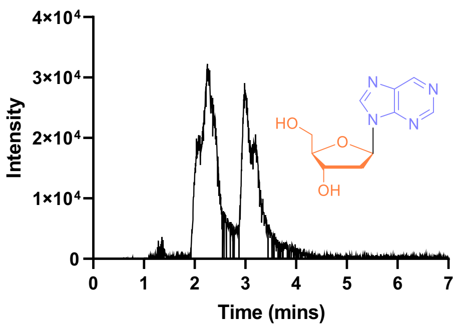

2'dAdo + purine

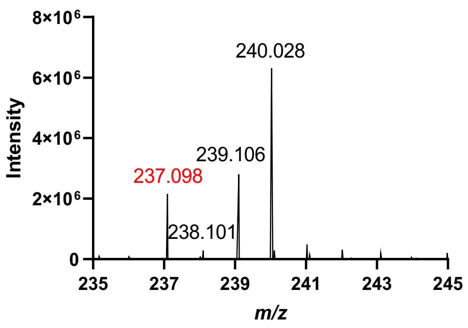

Product 2'-deoxy-3

2'dAdo + 2-fluoroadenine

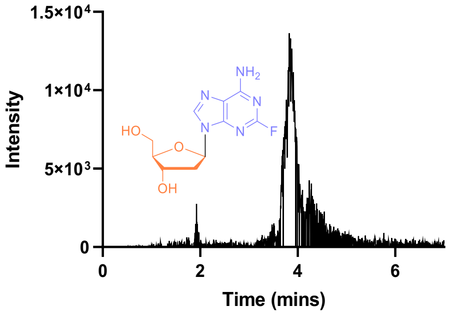

2'dAdo + 2-fluoroadenine

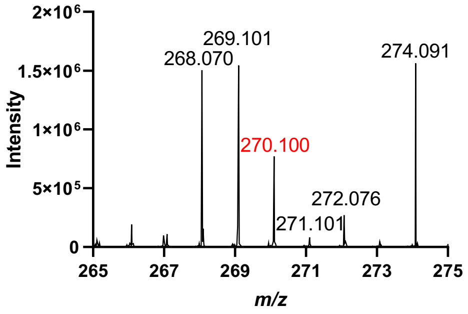

Product 2'-deoxy-4

Figure S1: Data for reactions to produce nucleosides.

2'dAdo + isoguanine

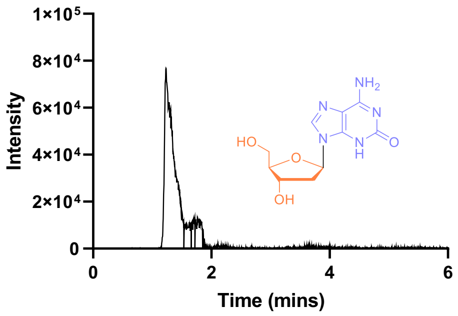

2'dAdo + isoguanine

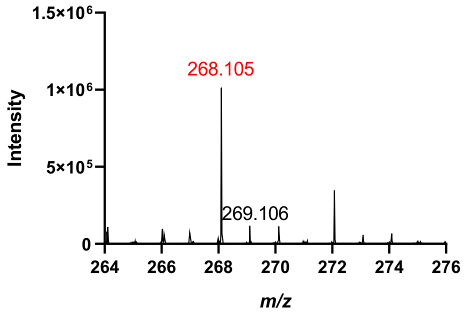

Product 2’-deoxy-5

2'dAdo + 1-methyl-3,7-dihydro-purine-2,6-dione

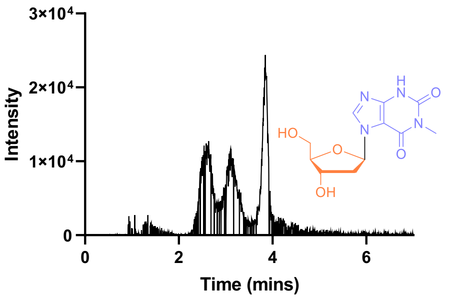

2'dAdo + 1-methyl-3,7-dihydro-purine-2,6-dione

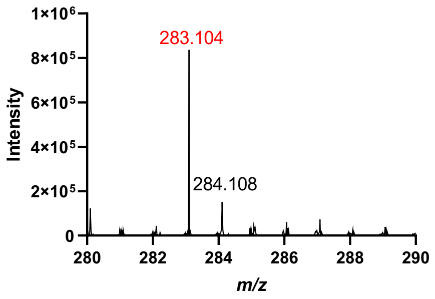

Product 2’-deoxy-6

2'dAdo + N-(7H-Purin-6-yl)benzamide

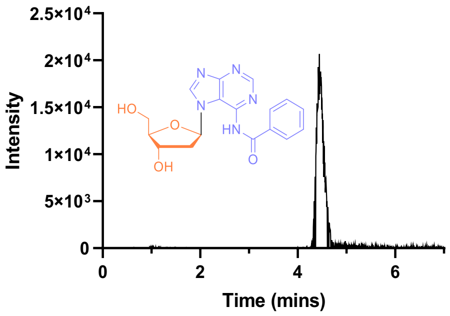

2'dAdo + N-(7H-Purin-6-yl)benzamide

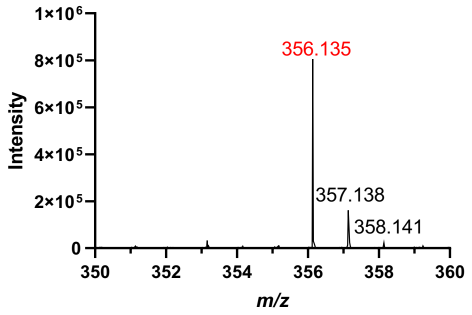

Product 2’-deoxy-7

2'dAdo + N,N-dimethyl-7H-purine-6-amine

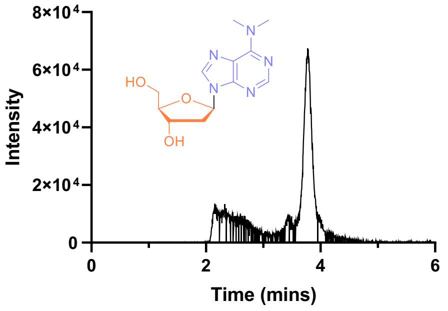

2'dAdo + N,N-dimethyl-7H-purine-6-amine

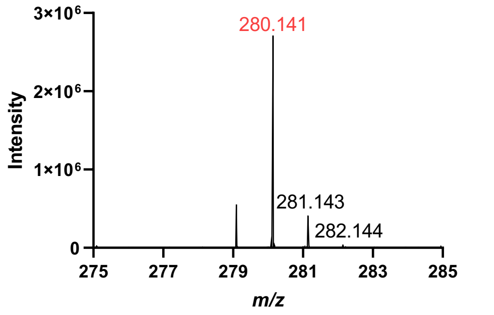

Product 2’-deoxy-8

Figure S1: Data for reactions to produce nucleosides.

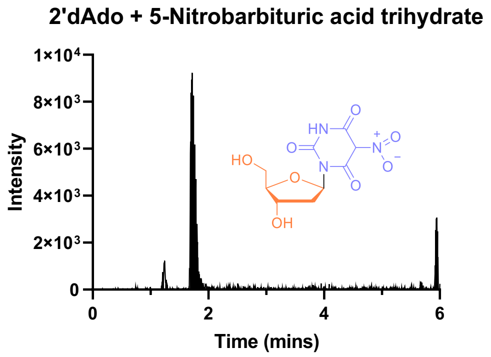

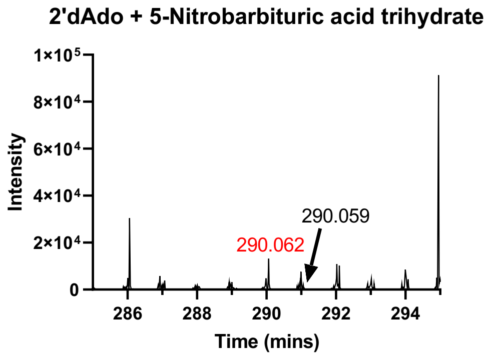

Product 2’-deoxy-9

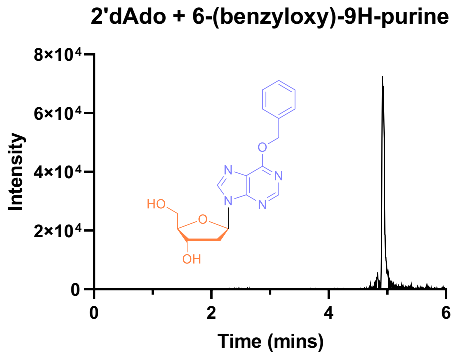

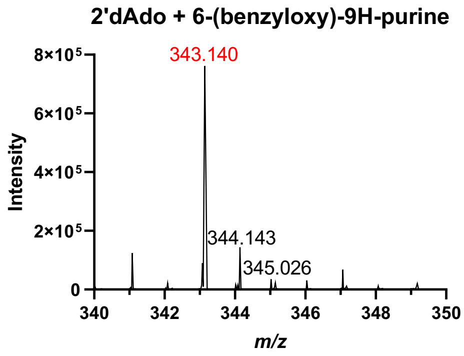

Product 2’-deoxy-11

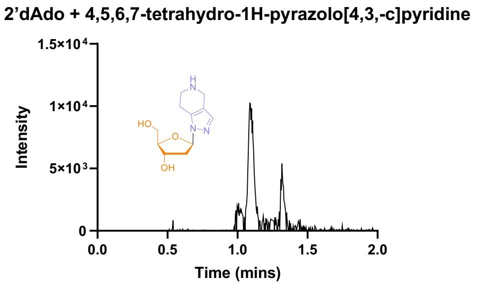

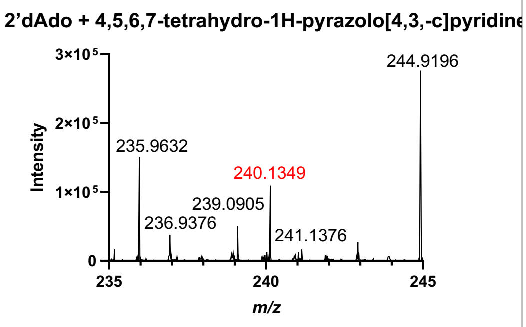

Product 2’-deoxy-12

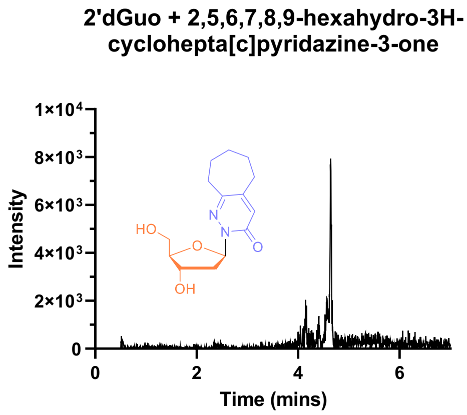

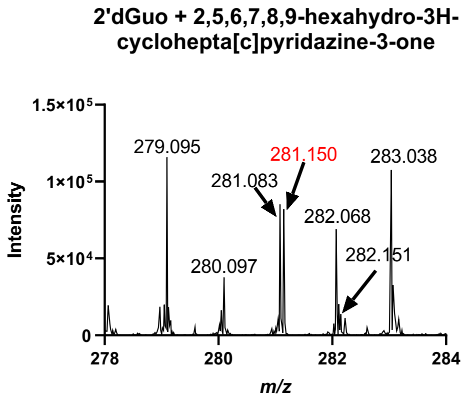

Figure S1: Data for reactions to produce nucleosides.

Product 2'-deoxy-13

2'dGuo + 1,4,5,6-Tetrahydropyrrolo [3,4-c]pyrazole dihydrochloride

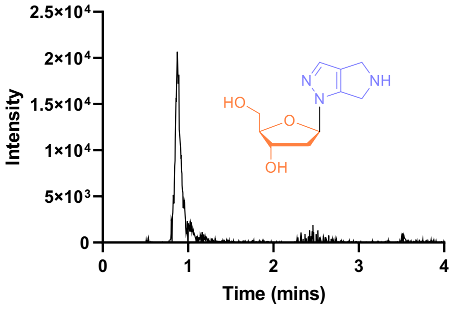

2'dGuo + 1,4,5,6-Tetrahydropyrrolo [3,4-c]pyrazole dihydrochloride

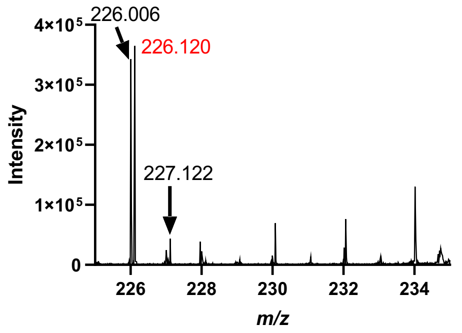

Product 2'-deoxy-14

2'dGuo + 5-chlorobenzotriazole

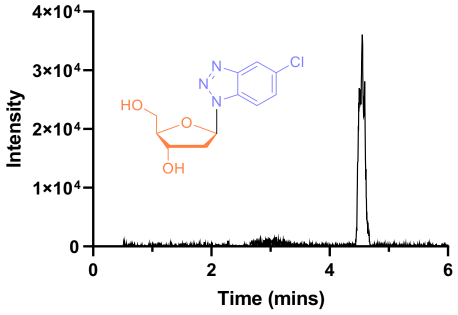

2'dGuo + 5-chlorobenzotriazole

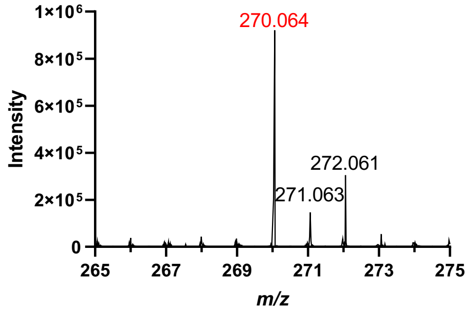

Product 2'-deoxy-15

2'dAdo + 6-mercaptopurine

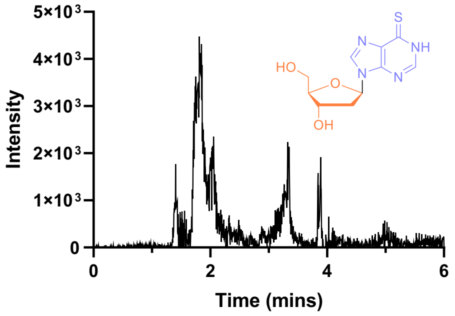

2'dAdo + 6-mercaptopurine

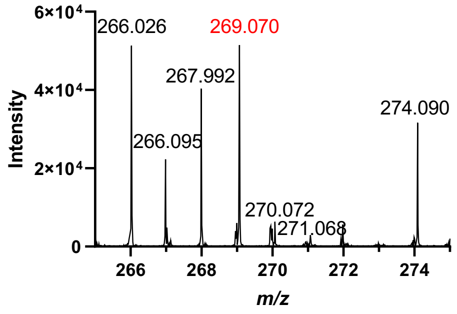

Product 2'-deoxy-16

Figure S1: Data for reactions to produce nucleosides.

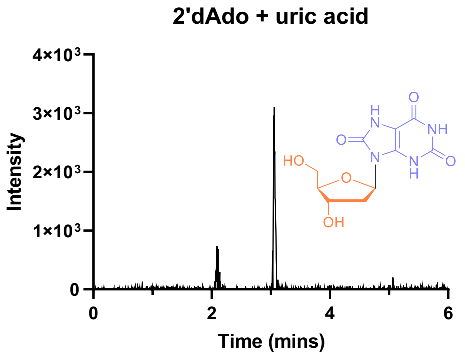

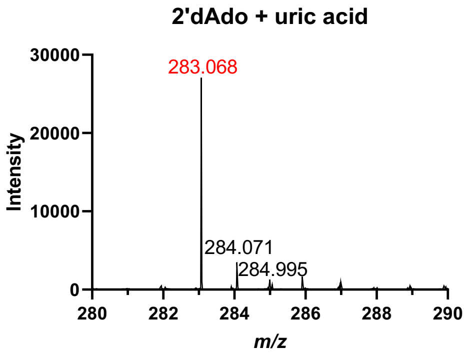

Product 2’-deoxy-19

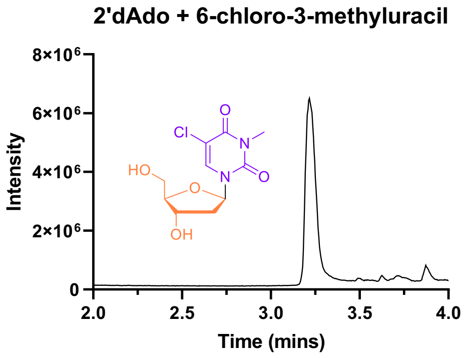

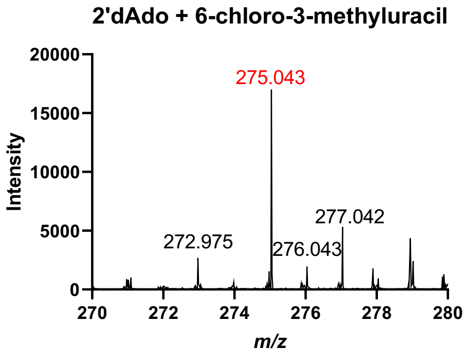

Product 2’-deoxy-21

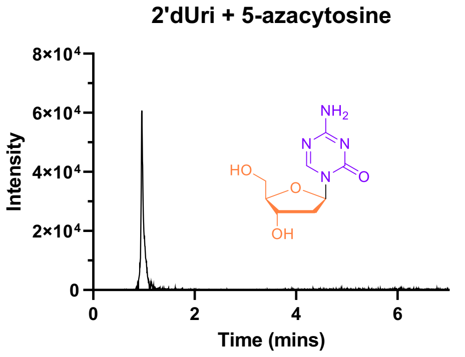

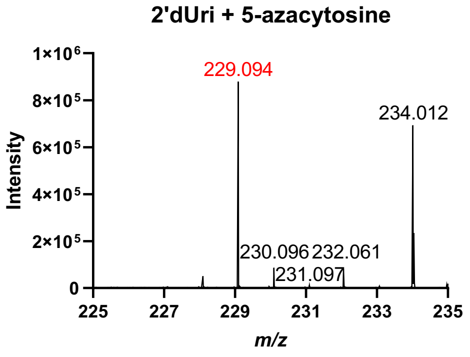

Product 2’-deoxy-22

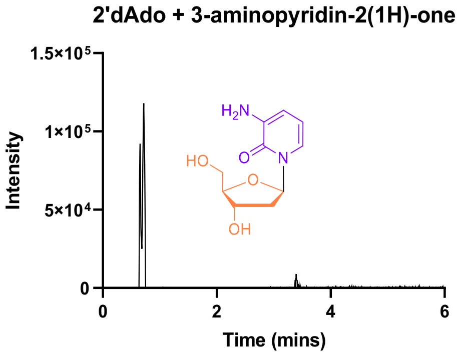

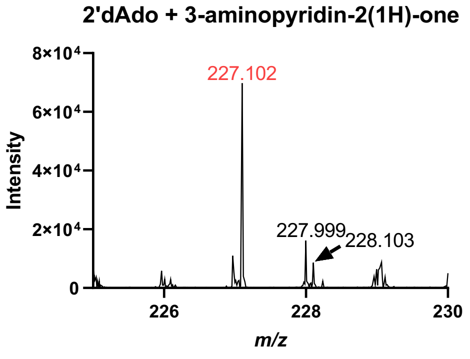

Product 2’-deoxy-38

Figure S1: Data for reactions to produce nucleosides.

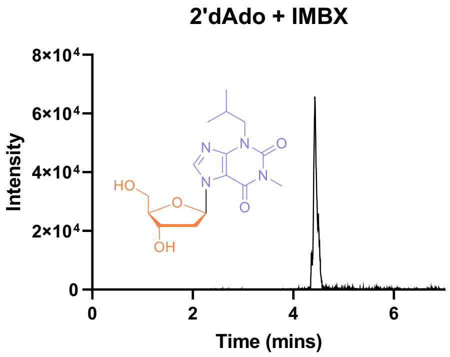

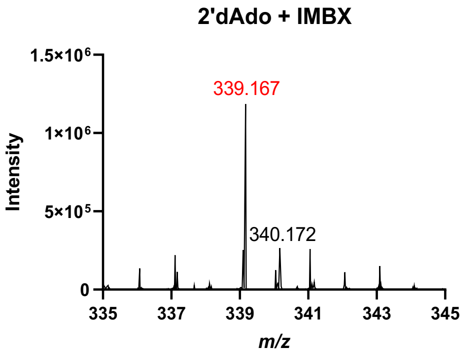

Product 2’-deoxy-47

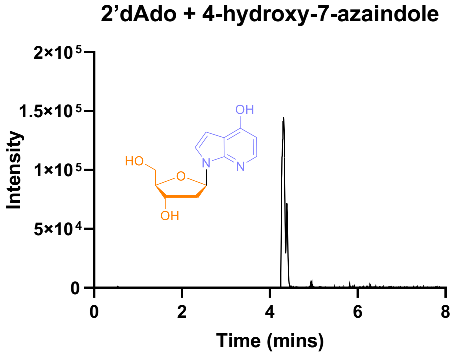

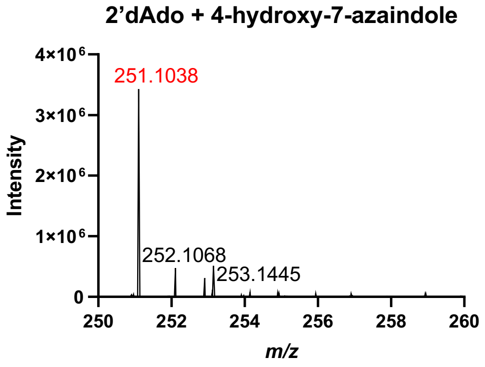

Product 2’-deoxy-48

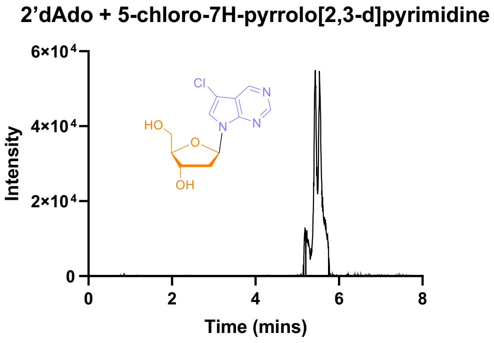

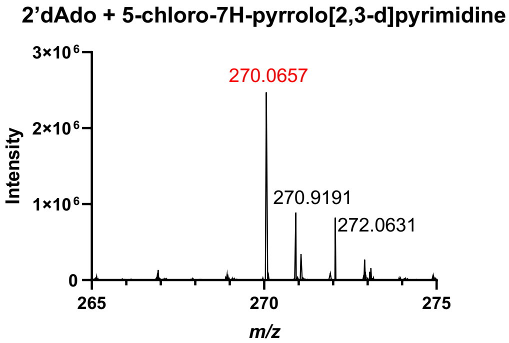

Product 2’-deoxy-54

Figure S1: Data for reactions to produce nucleosides.

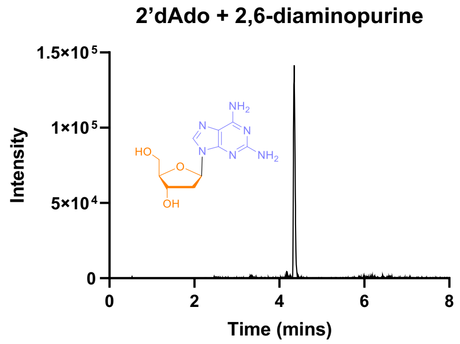

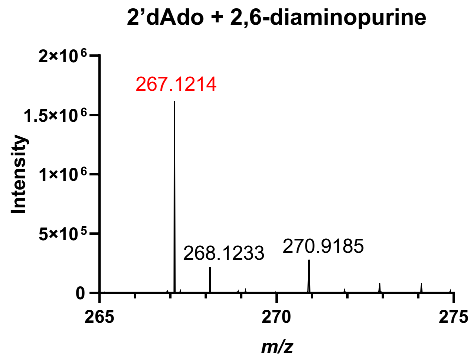

Standard nucleobase substrates

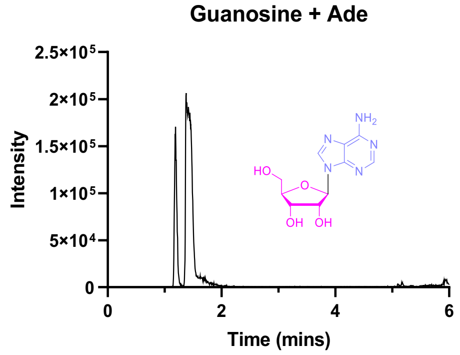

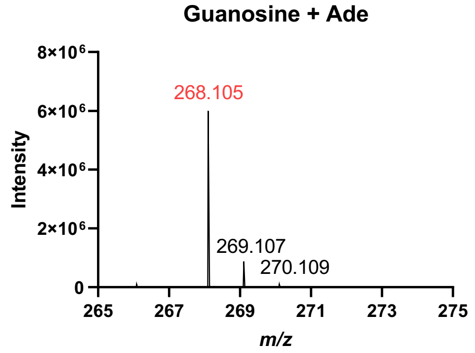

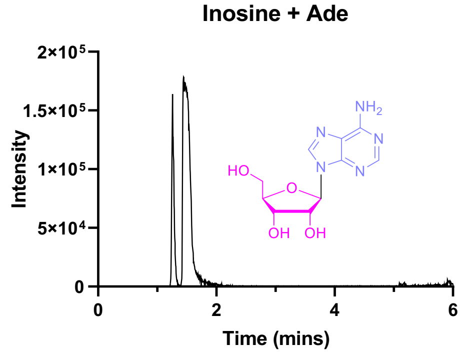

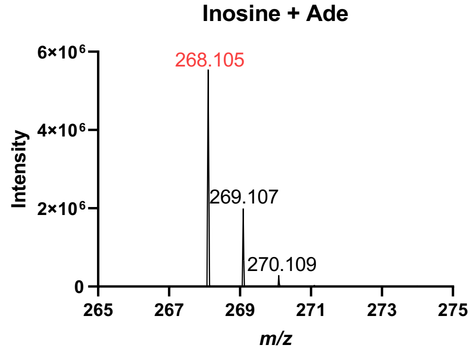

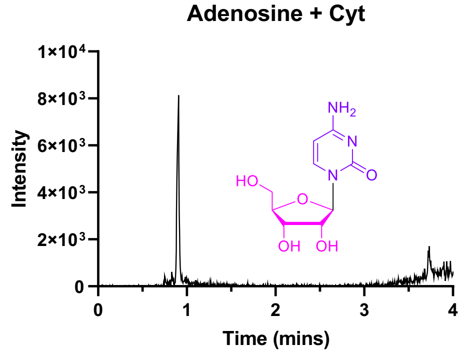

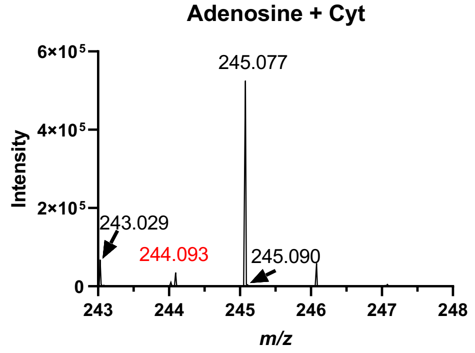

2'-amino-2'-deoxy-adenosine

Figure S1: Data for reactions to produce nucleosides.

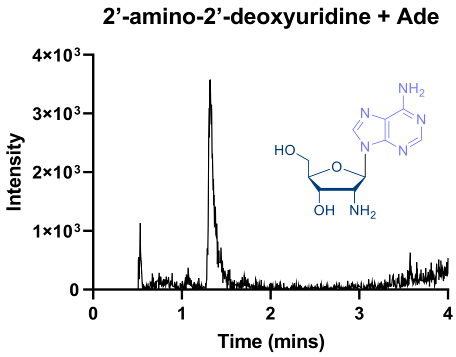

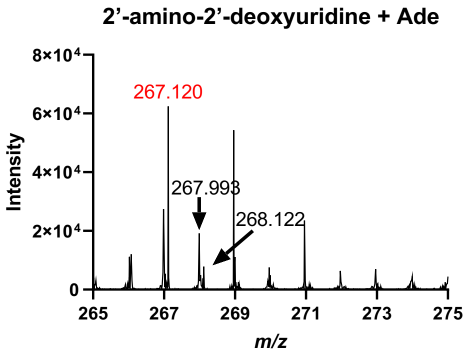

3'-deoxy-adenosine (cordycepin)

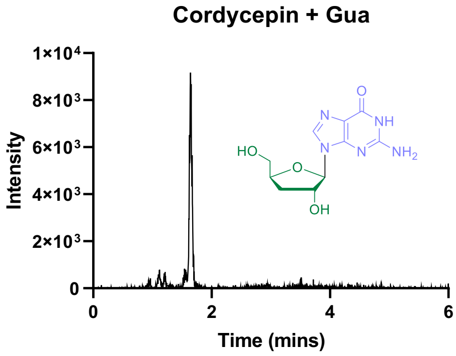

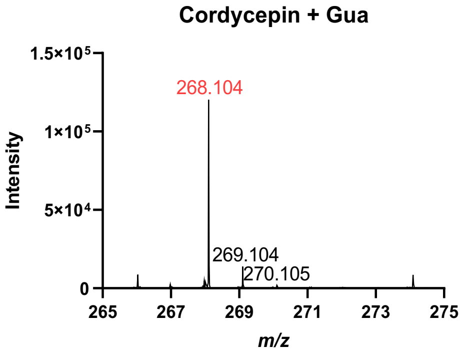

CtNDT Y7F – confirmation of activity with ribonucleosides

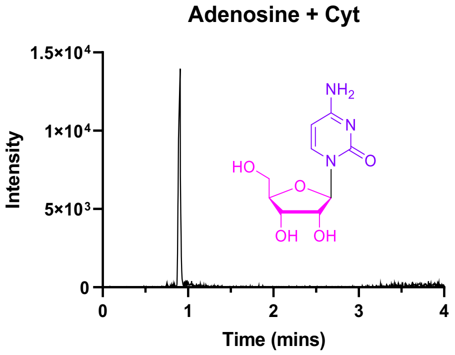

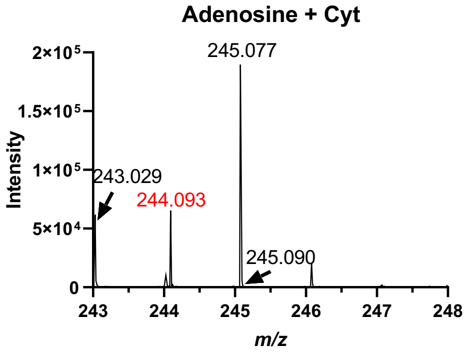

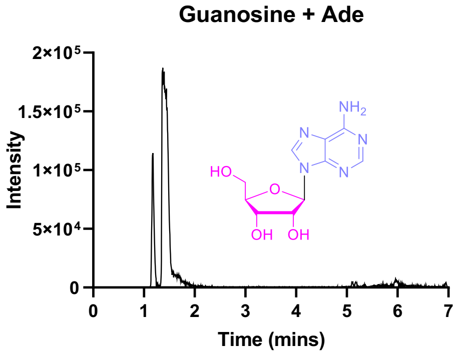

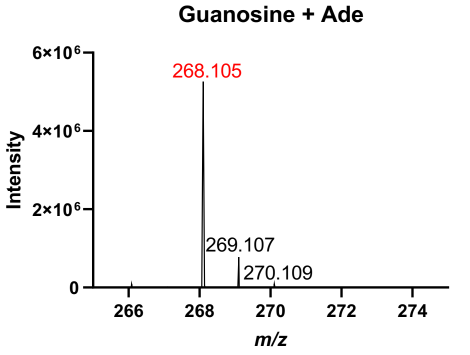

Figure S1: Data for reactions to produce nucleosides.

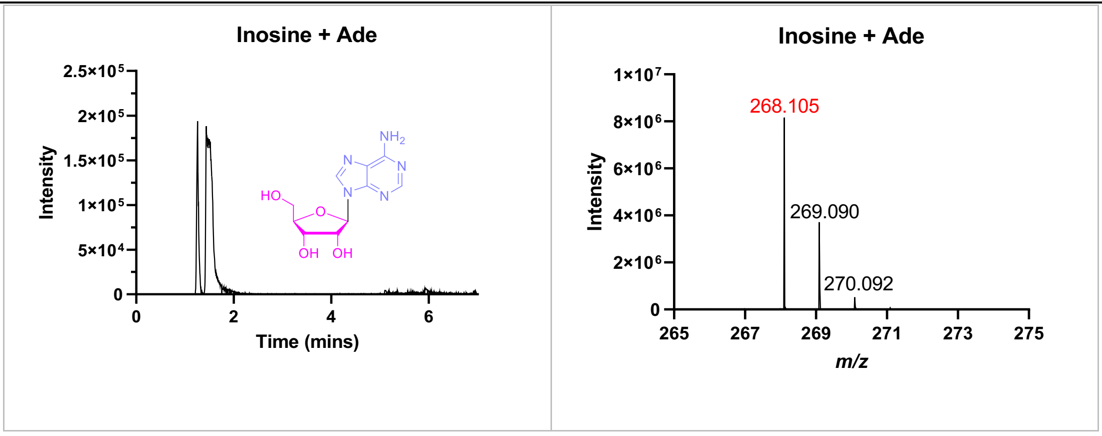

Figure S1: Data for reactions to produce nucleosides.

CtNDT$_{\text{Y7F-A9S}}$

Product 3’-deoxy-2

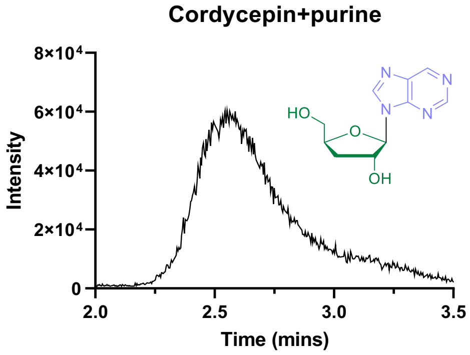

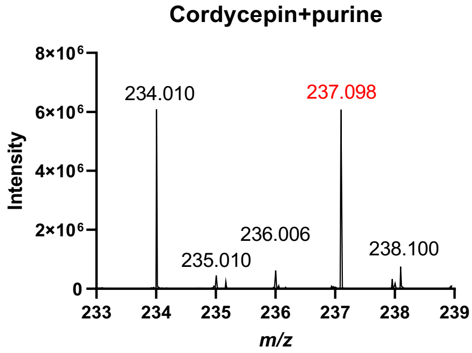

Product 3’-deoxy-3

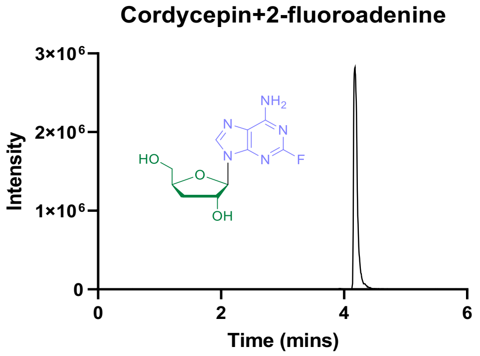

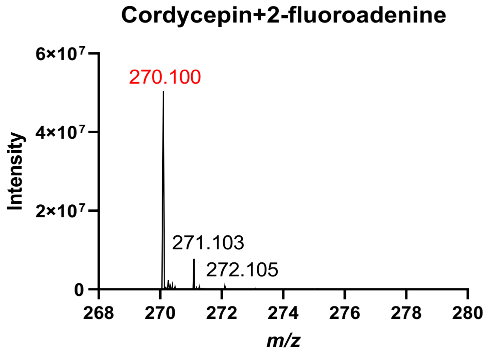

Product 3’-deoxy-5

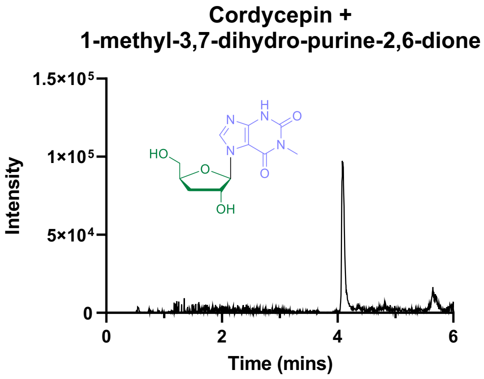

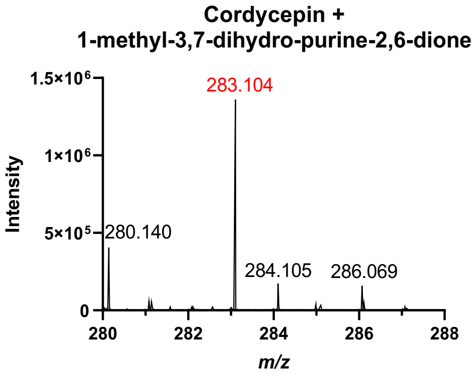

Figure S1: Data for reactions to produce nucleosides.

Product 3'-deoxy-7

Cordycepin +
N,N-dimethyl-7H-purine-6-amine

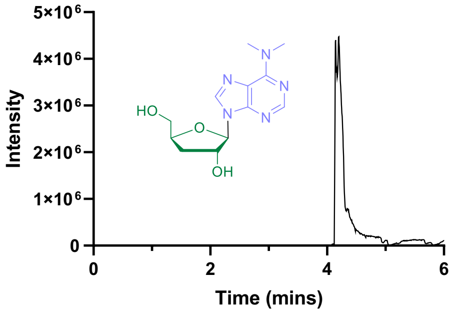

Cordycepin +
N,N-dimethyl-7H-purine-6-amine

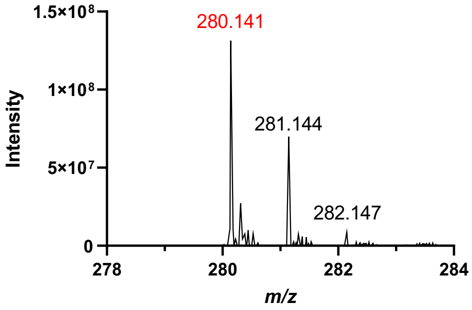

Product 3'-deoxy-9

Cordycepin+6-(benzyloxy)-9H-purine

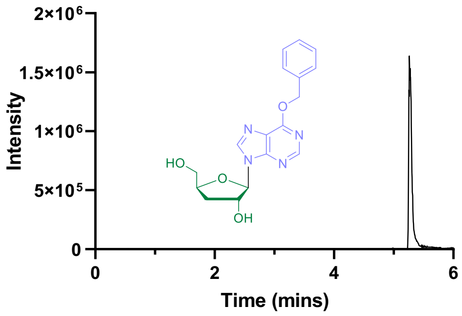

Cordycepin+6-(benzyloxy)-9H-purine

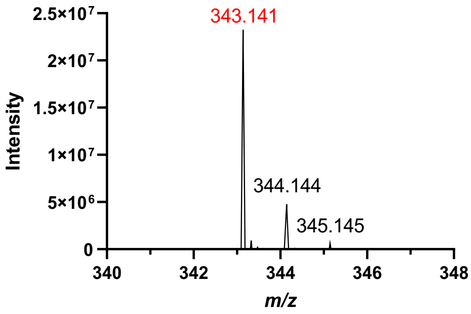

Product 3'-deoxy-10

Cordycerpin+1H-Indazole

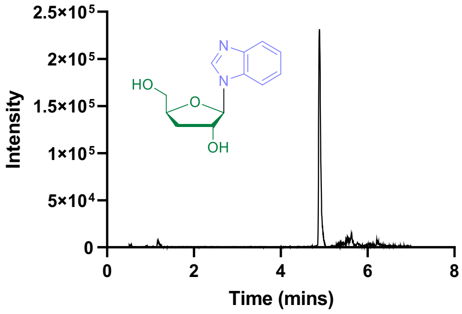

Cordycerpin+1H-Indazole

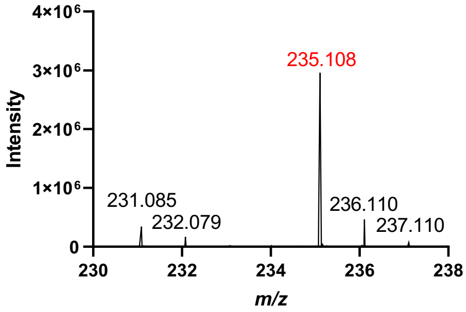

Figure S1: Data for reactions to produce nucleosides.

Product 3’-deoxy-15

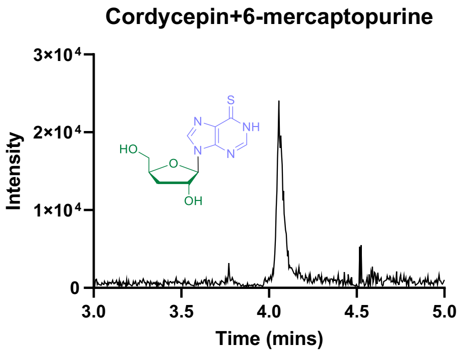

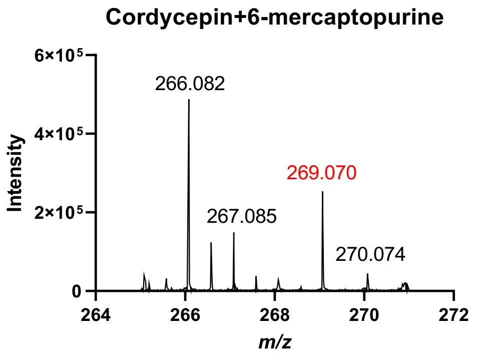

Product 3’-deoxy-38

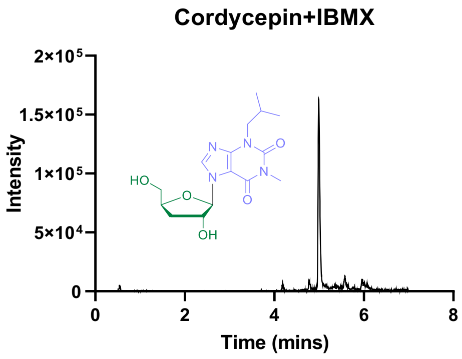

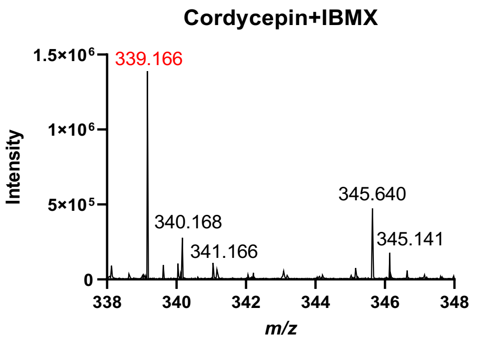

Product 3’-deoxy-54

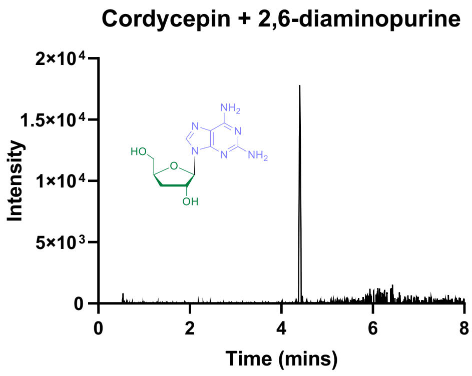

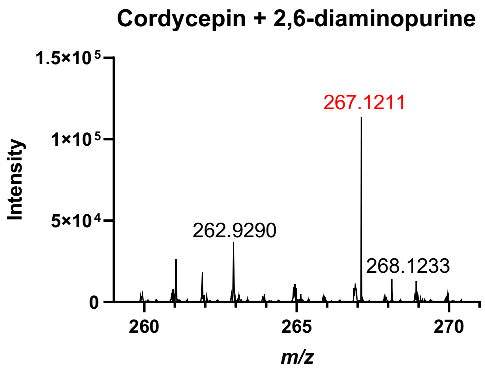

Product ribo-1

Figure S1: Data for reactions to produce nucleosides.

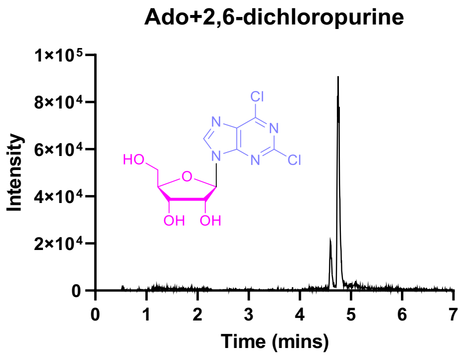

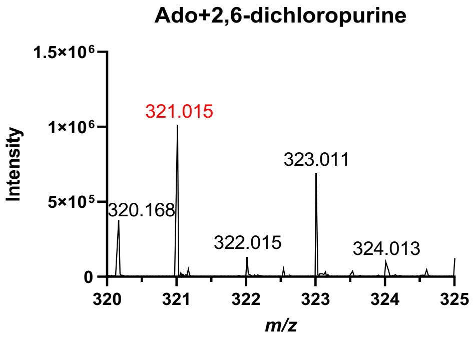

Product ribo-2

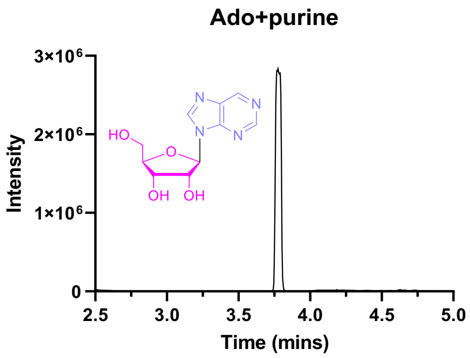

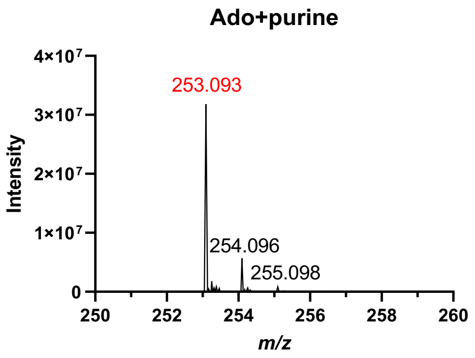

Product ribo-3

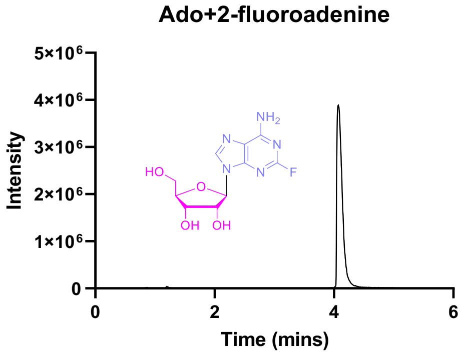

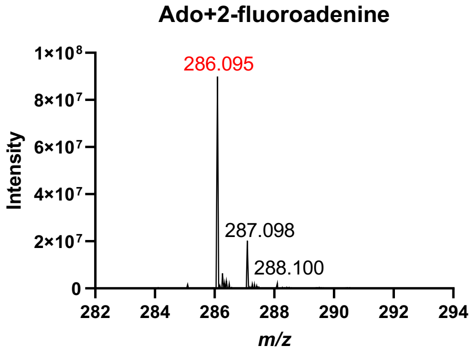

Product ribo-5

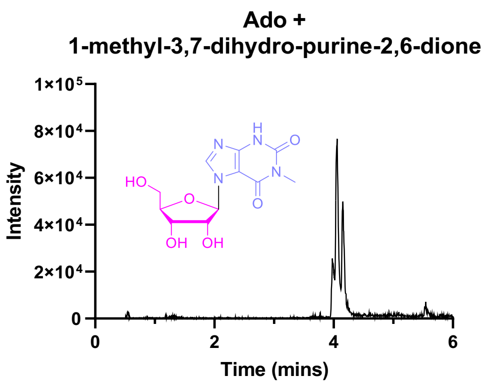

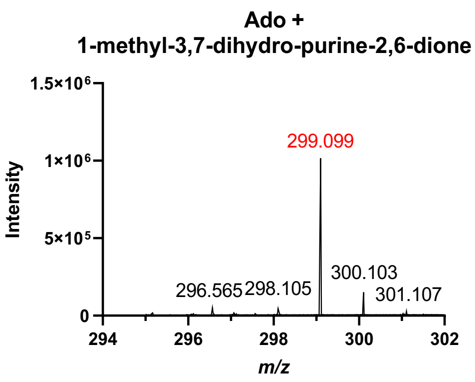

Figure S1: Data for reactions to produce nucleosides.

Product ribo-6

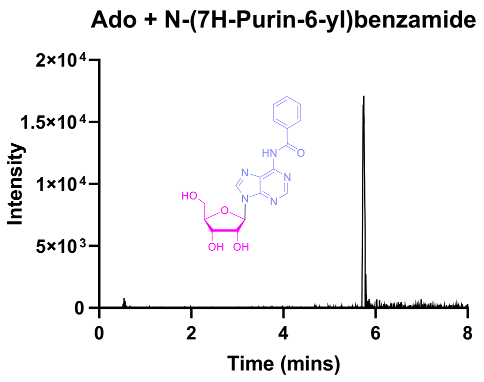

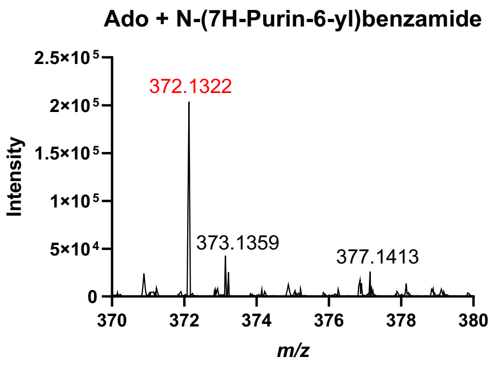

Product ribo-7

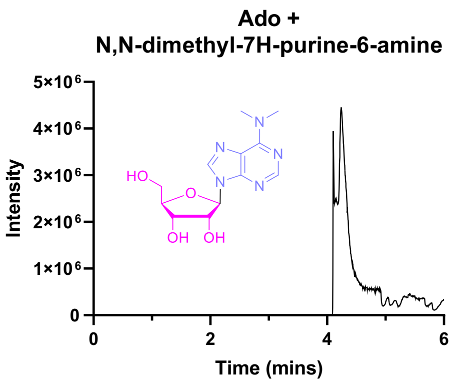

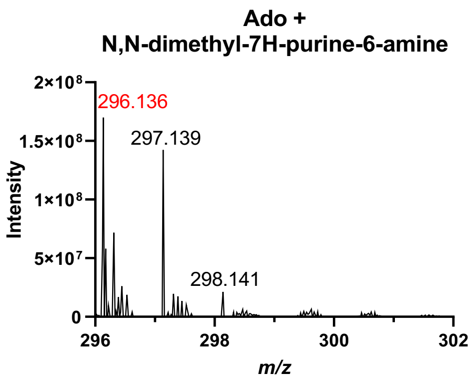

Product ribo-9

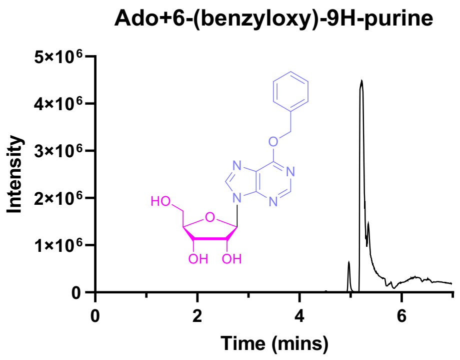

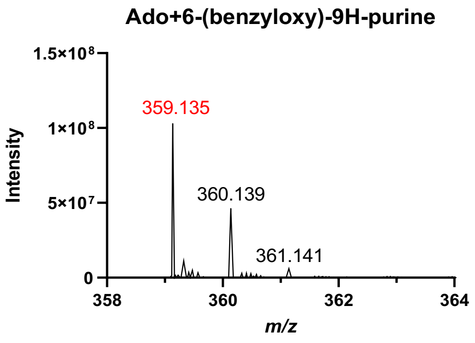

Product ribo-10

Figure S1: Data for reactions to produce nucleosides.

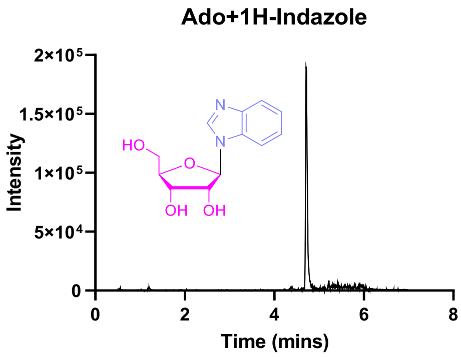

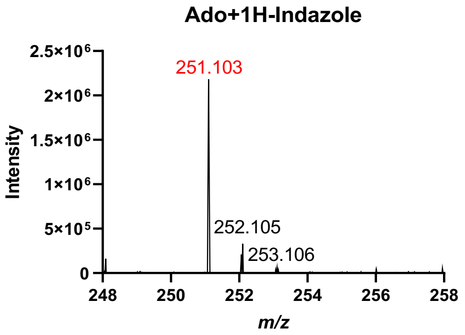

Product ribo-13

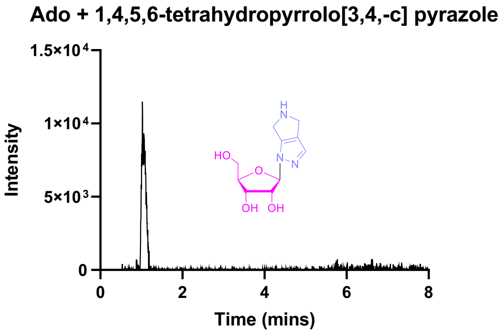

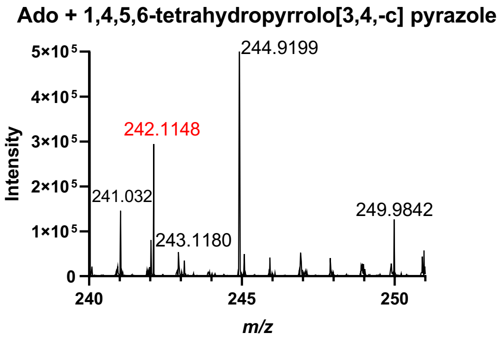

Product ribo-14

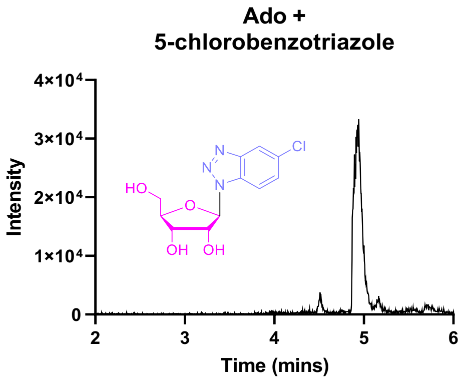

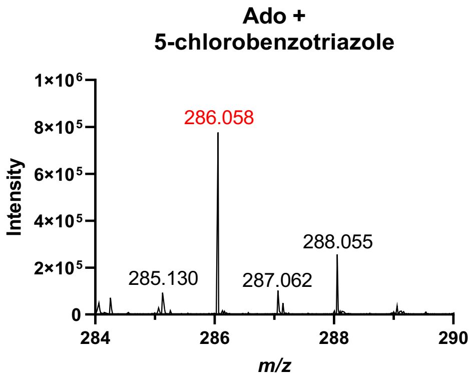

Product ribo-15

Figure S1: Data for reactions to produce nucleosides.

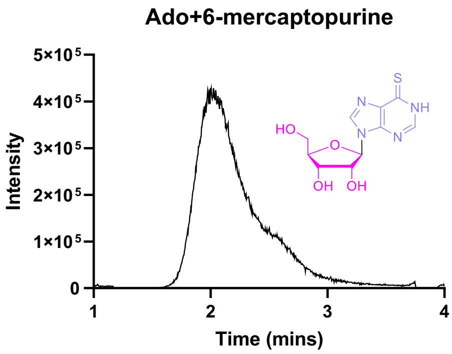

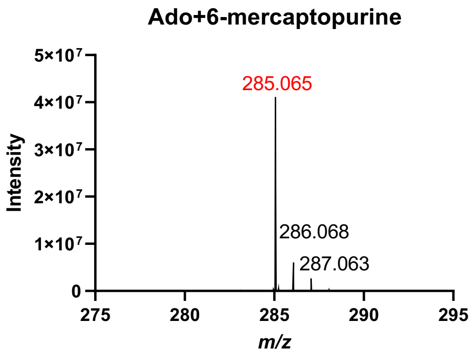

Product ribo-38

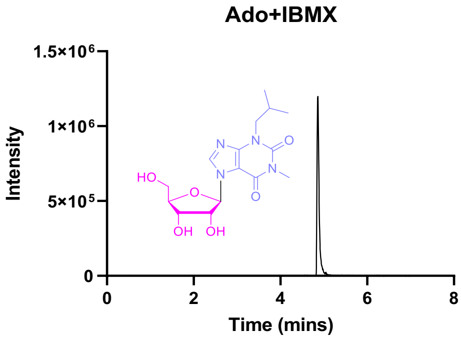

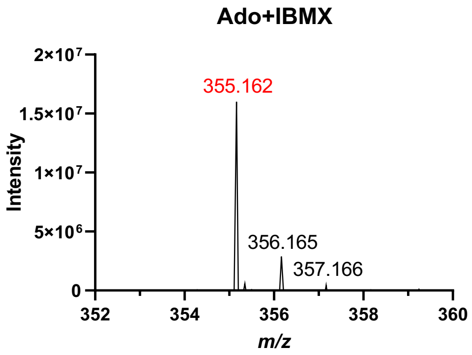

Product ribo-47

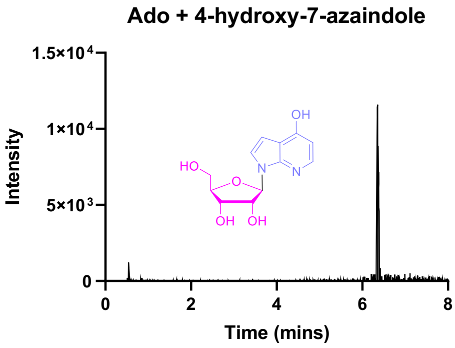

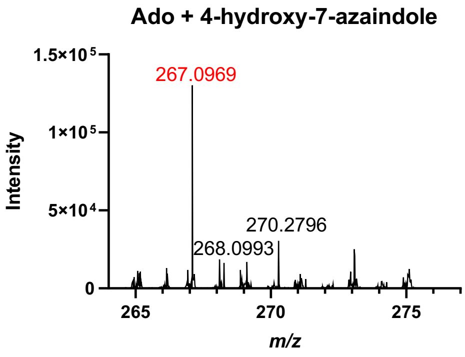

Product ribo-54

Figure S1: Data for reactions to produce nucleosides.

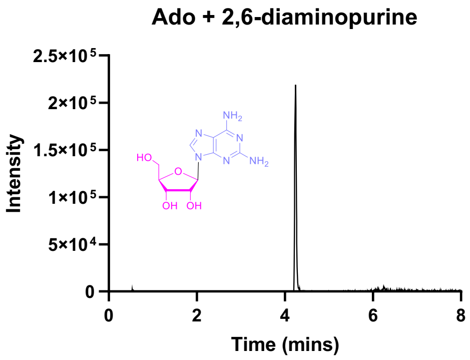

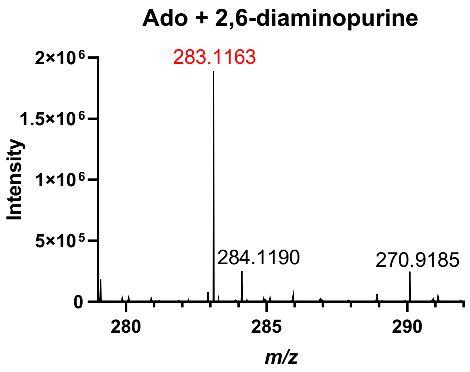

BpNDT

Product 2’-deoxy-18

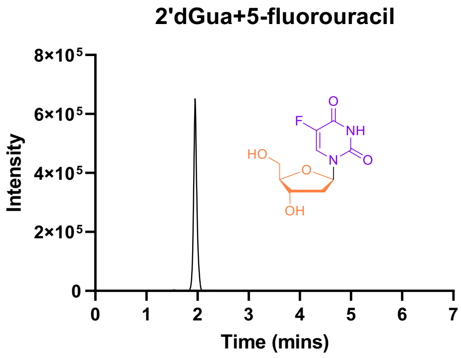

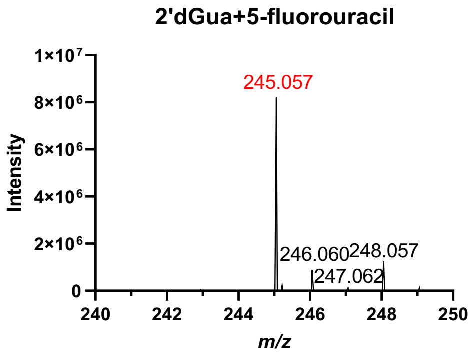

Product 2’-deoxy-20

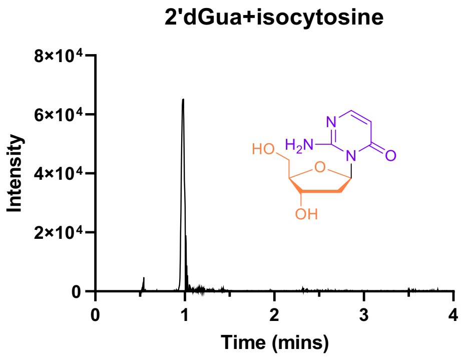

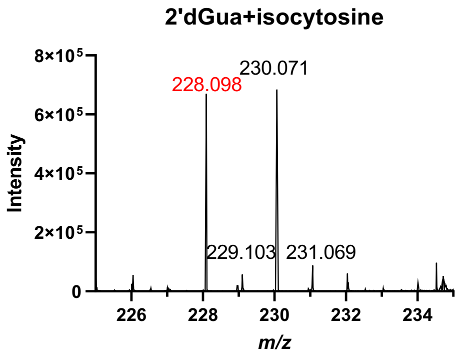

Product 2’-deoxy-21

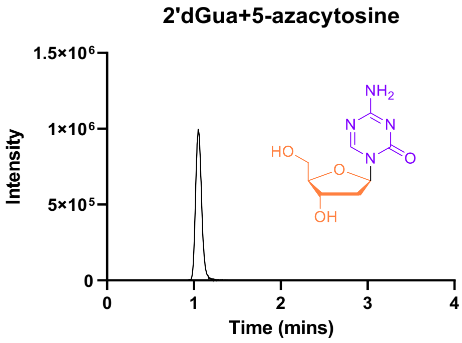

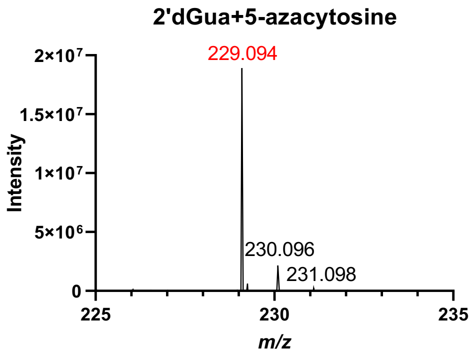

Figure S1: Data for reactions to produce nucleosides.

Product 2’-deoxy-23

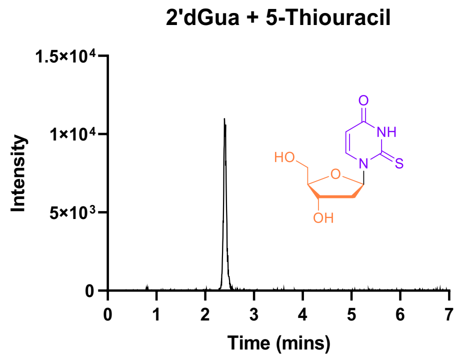

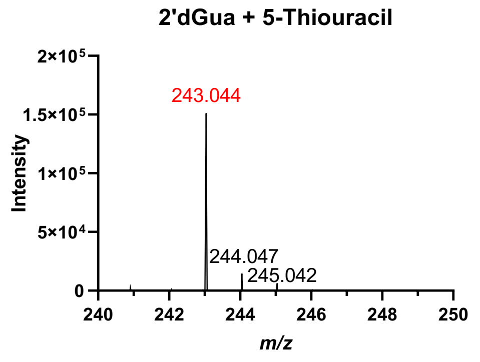

Product 2’-deoxy-24

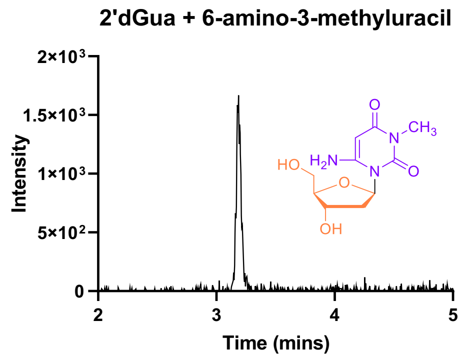

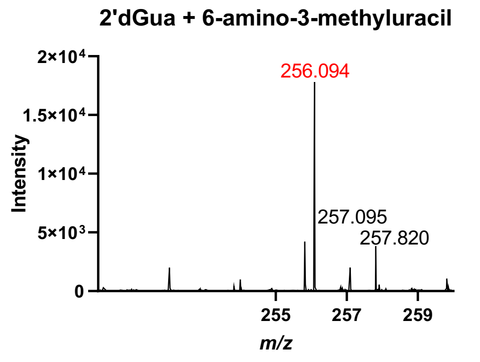

Product 2’-deoxy-26

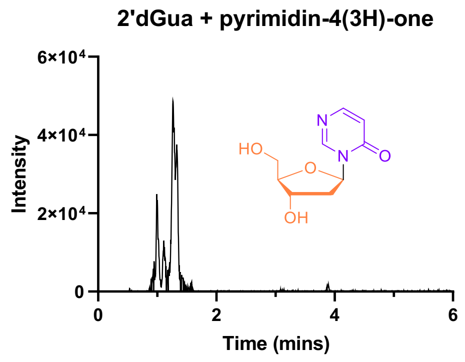

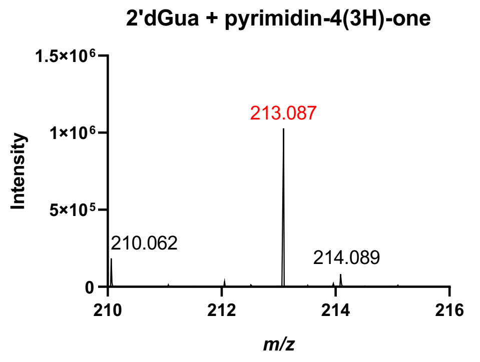

Product 2’-deoxy-29

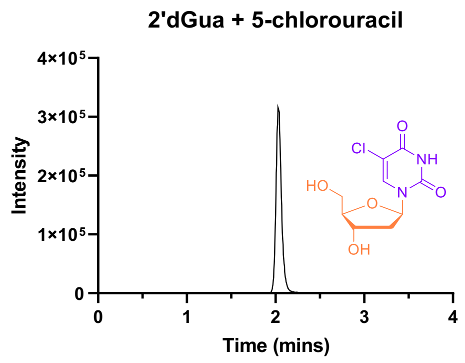

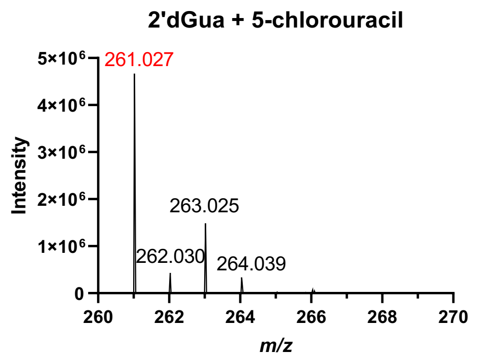

Product 2’-deoxy-30

Figure S1: Data for reactions to produce nucleosides.

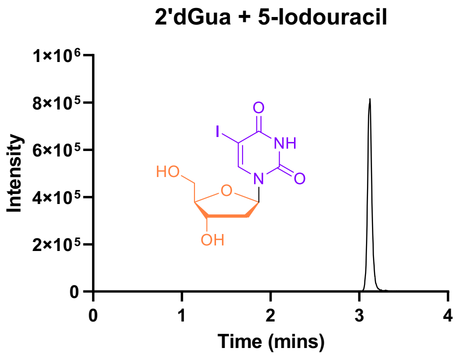

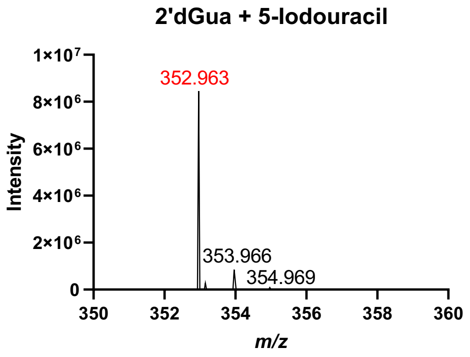

Product 2’-deoxy-32

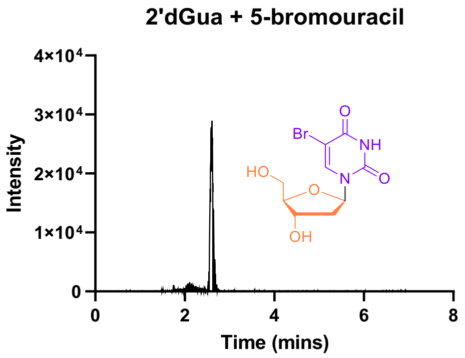

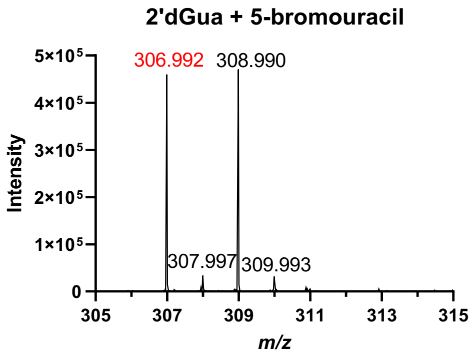

Product 2’-deoxy-43

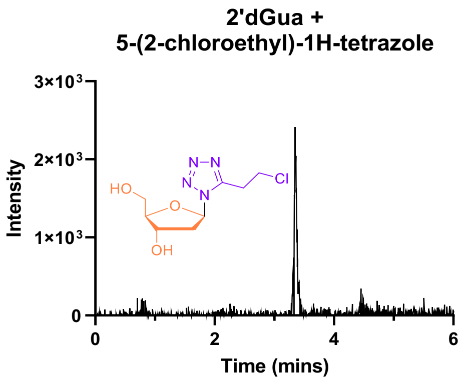

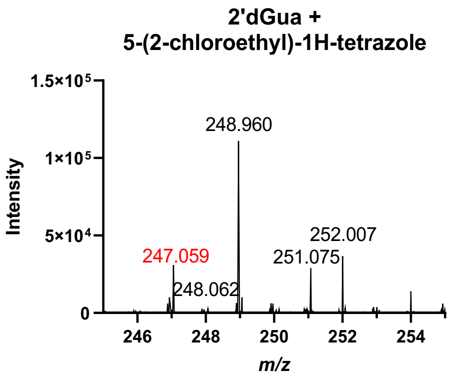

BpNDT Y5F

Product ribo-18

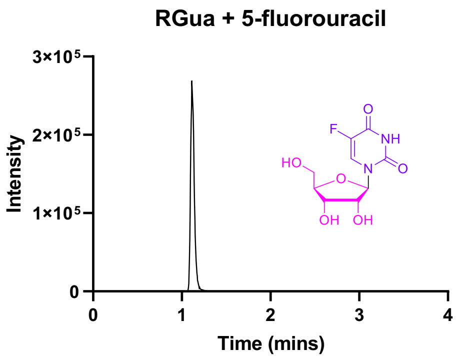

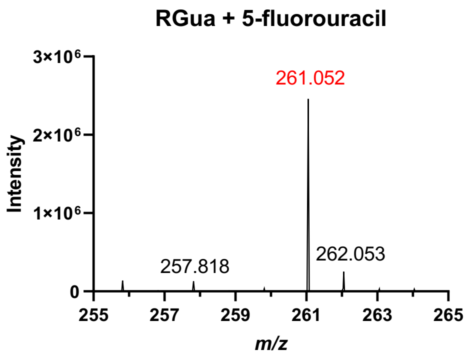

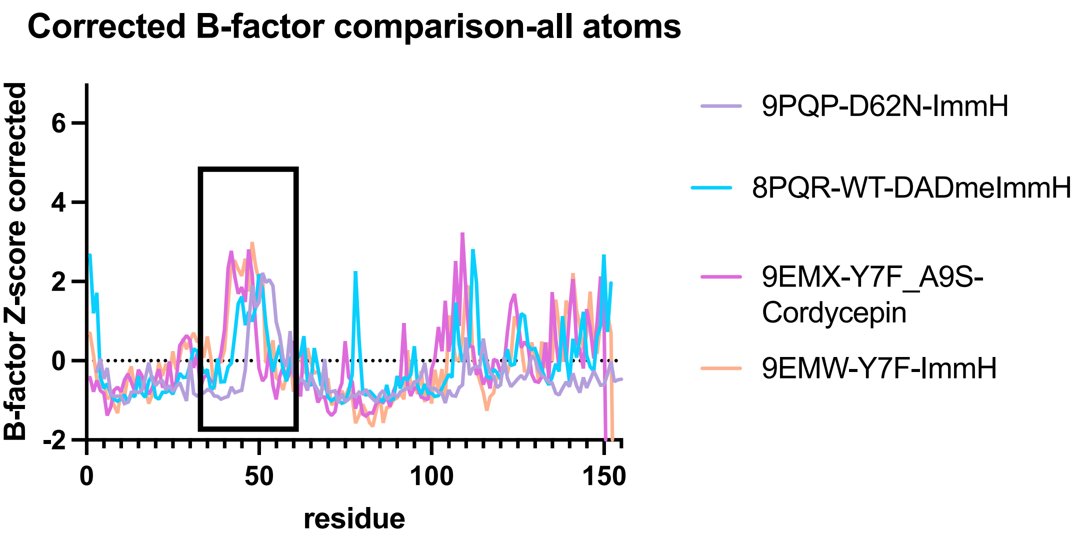

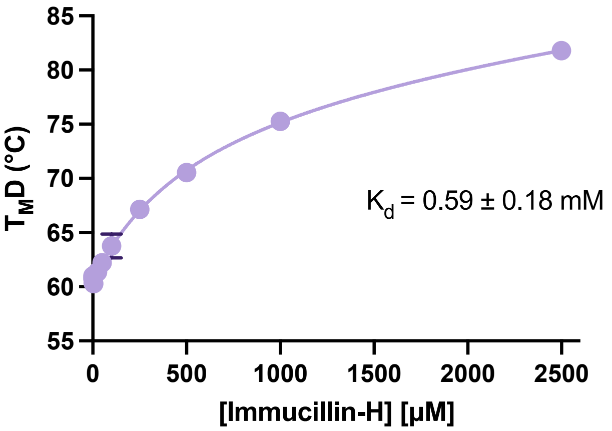

Figure S2: Interactions of *Ct*NDT with transition state analogue DADmeImmH and CtNDT$_{Y7F\text{-}A9S}$ with substrate analogue ImmH. Top: Comparison between apo and CtNDT bound to transition state analogue DADmeImmH1, depicting a region of higher B-factors in the apo enzyme which undergoes a small degree of stabilization upon tight binding of this analogue. Corrected B-factors ploted were obtained using Baverage from the CCP4 suite[6] and the correction previously described[7]. The correction is needed to compare B-factors as they can be affected by resolution and refinement strategies. Bottom: Binding curve for *Ct*NDT$_{Y7F\text{-}A9S}$ and Immucillin-H, carried out using differential scanning fluorimetry.

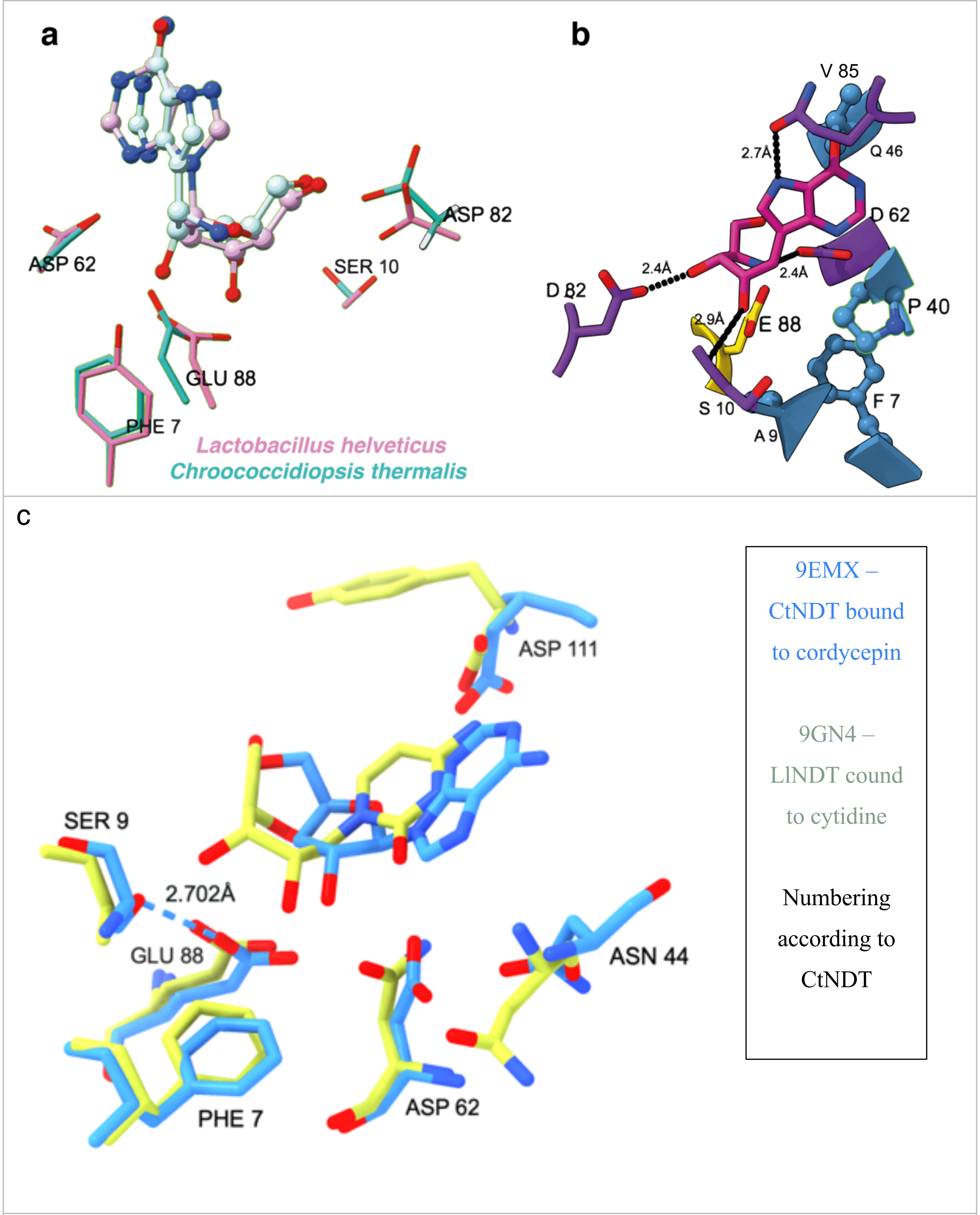

d

		Area (SA) Å2	Volume (SA) Å3
Enzyme	pdb	Pocket area	Pocket volume
Chroococcidiopsis thermalis	8PQQ	251.93	149.949
Trypanosoma brucei	2A0K	264.734	147.782
Lactobacillus leichmannii	1F8X	148.517	170.618

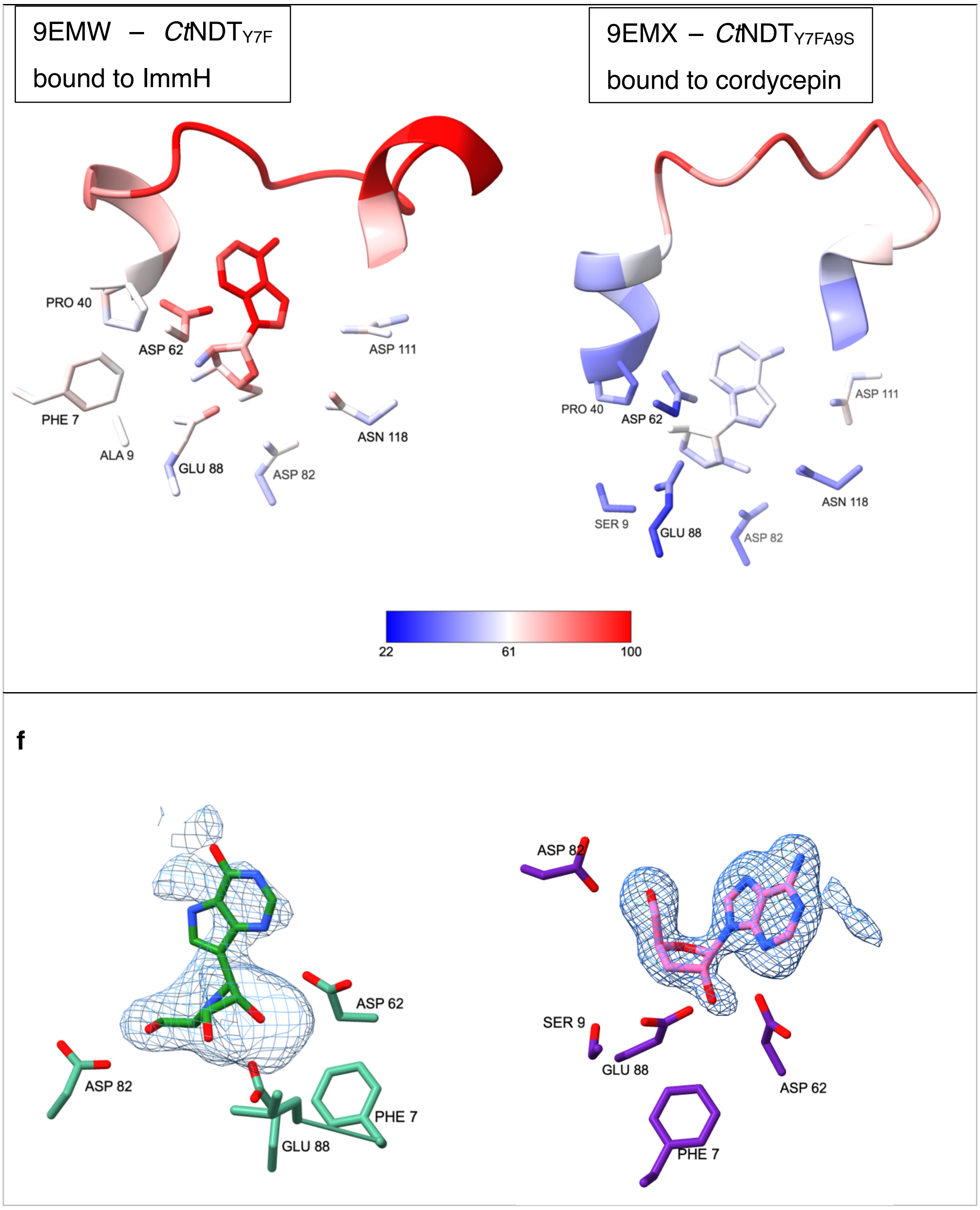

Figure S3: Ligand binding site of NDT enzymes. a and b, residues interacting with ribosyl moiety of nucleoside susbtrates. a) Comparison between *Lactobacillus helveticus* (pink, 1s2g[8], complex with 2'deoxyadenosine, rmsd 0.89 Å) and CtNDT$_{Y7F}$ (blue, this work, pdb 9EMW, complex with ImmH). b) Distances between key residues in the binding pocket for CtNDT$_{Y7F}$ interacting with ImmH. d) Comparison between substrate binding pocket volumes of different NDT enzymes. e) Residues and ligand atoms colored by normalized B factor (using Baverage -

https://www.ccp4.ac.uk/html/baverage.html), where smaller B factors are in blue and larger B factors are in red as per the color key in the bottom of the figure. f) Likelihood weighted mFo-DFc omit map at 2.5 σ contour (generated using Phenix maps), left 9EMW – *Ct*NDT$_{Y7F}$ bound to ImmH, right 9EMX – *Ct*NDT$_{Y7FA9S}$ bound to cordycepin).

Figure S4. HPLC and raw kinetic data for all enzyme variants tested.

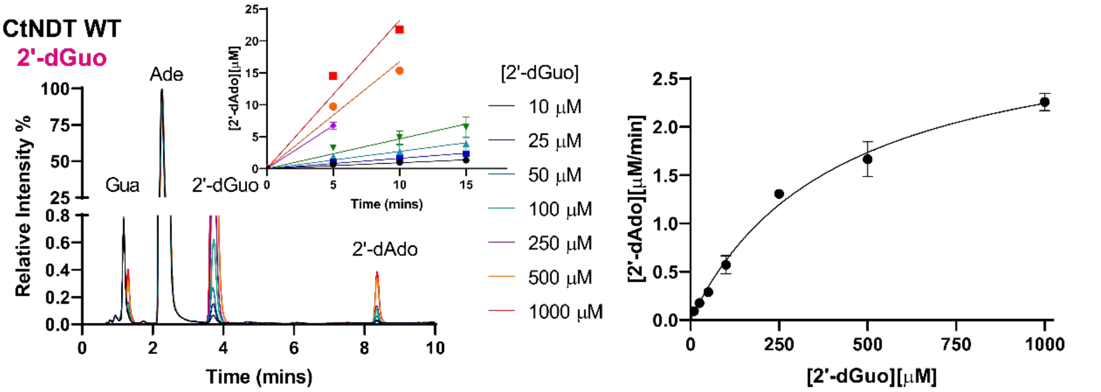

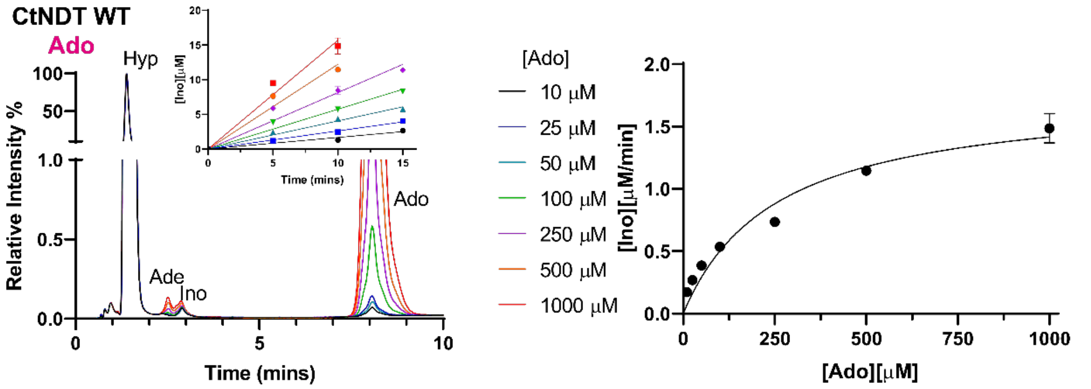

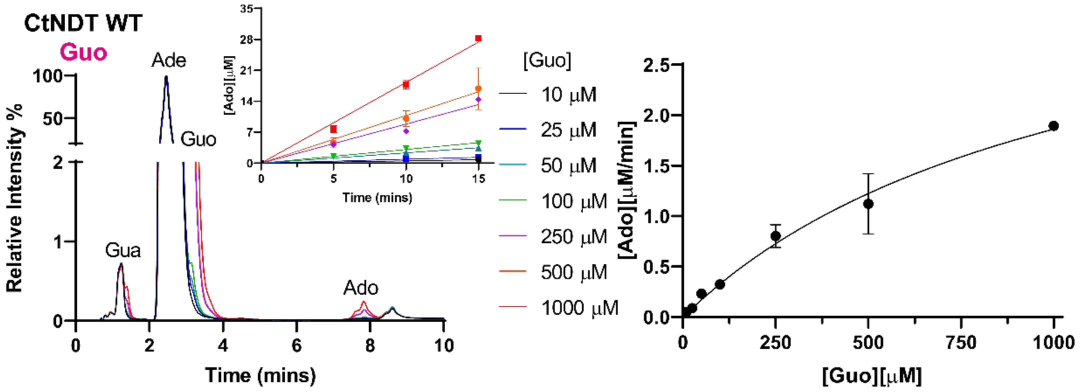

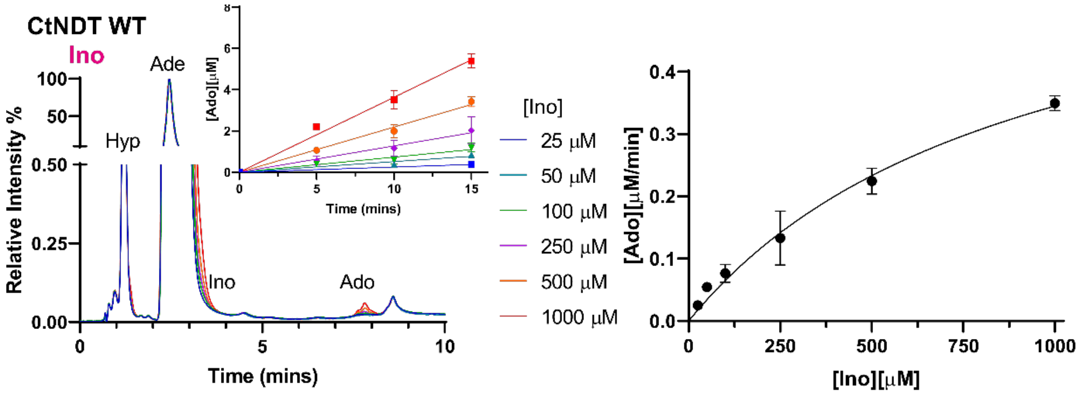

Figure S4. HPLC and raw kinetic data for all enzyme variants tested.

CtNDT WT

Uric acid

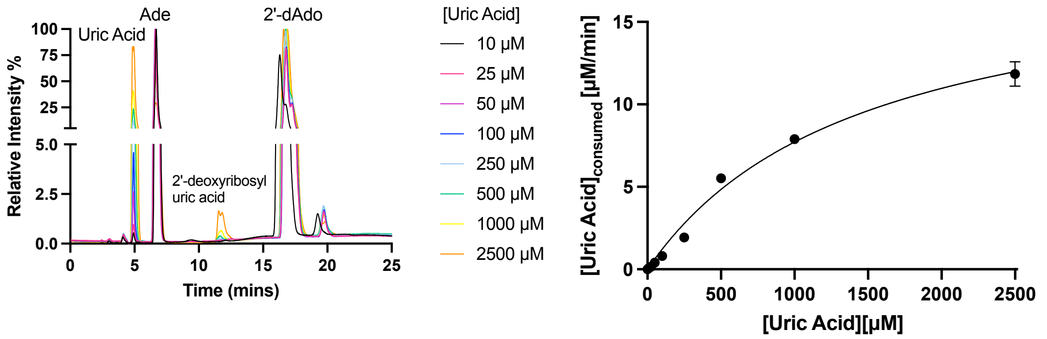

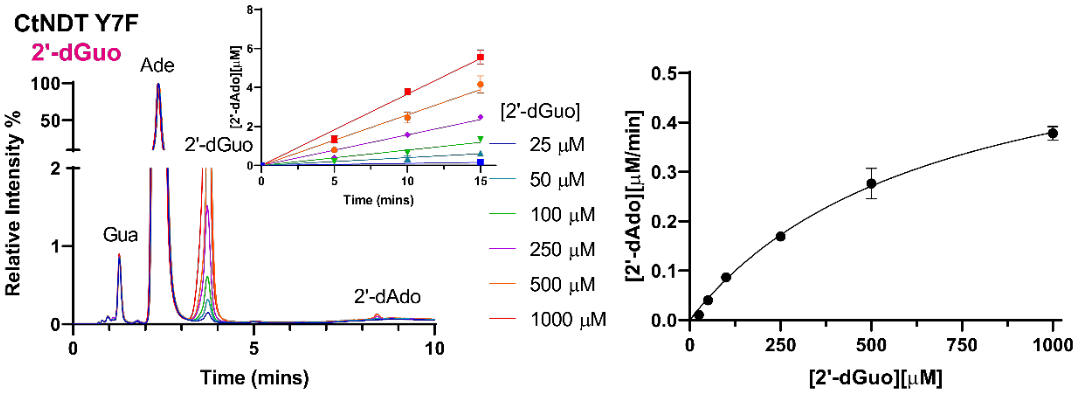

Figure S4. HPLC and raw kinetic data for all enzyme variants tested.

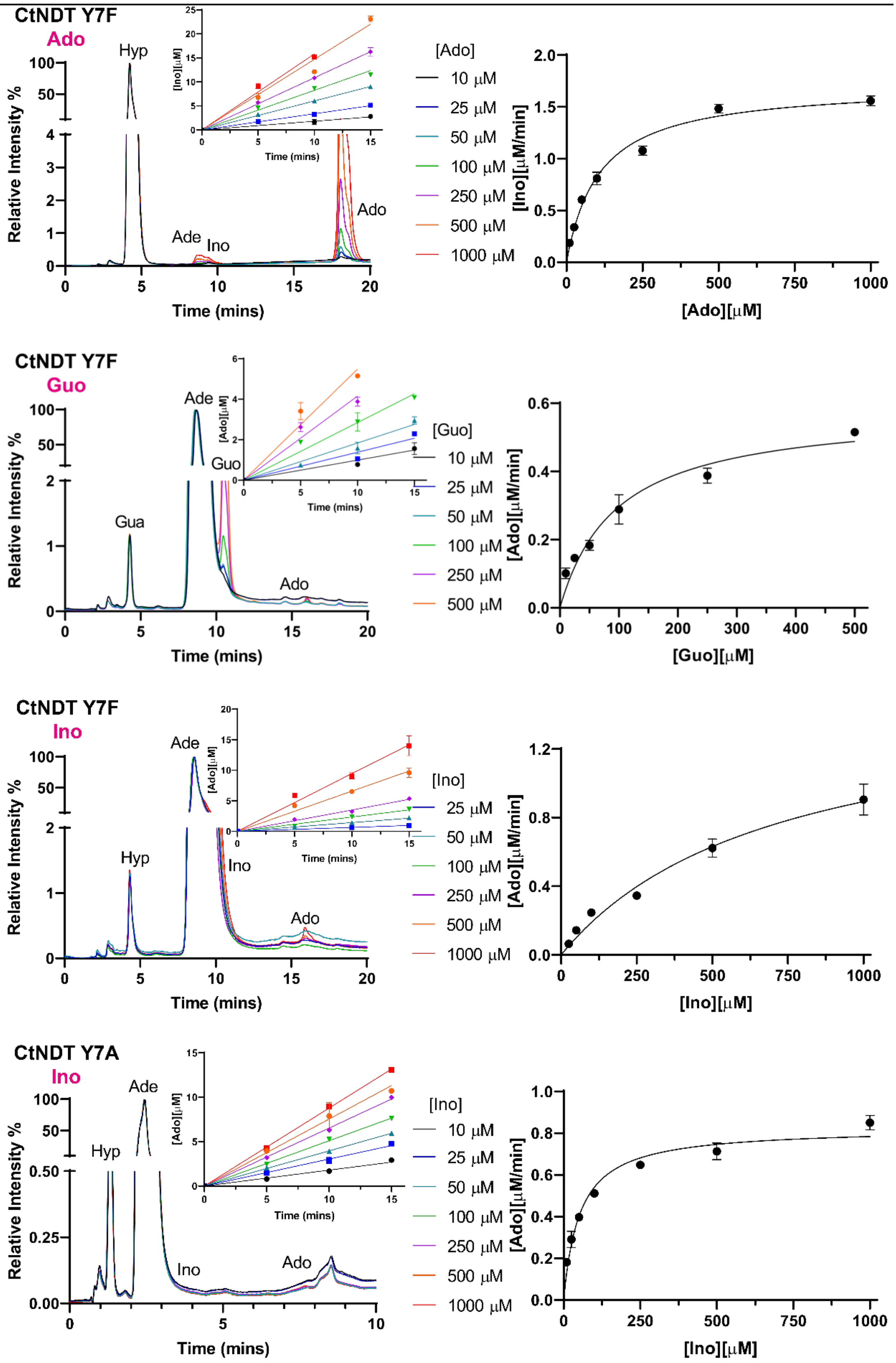

Figure S4. HPLC and raw kinetic data for all enzyme variants tested.

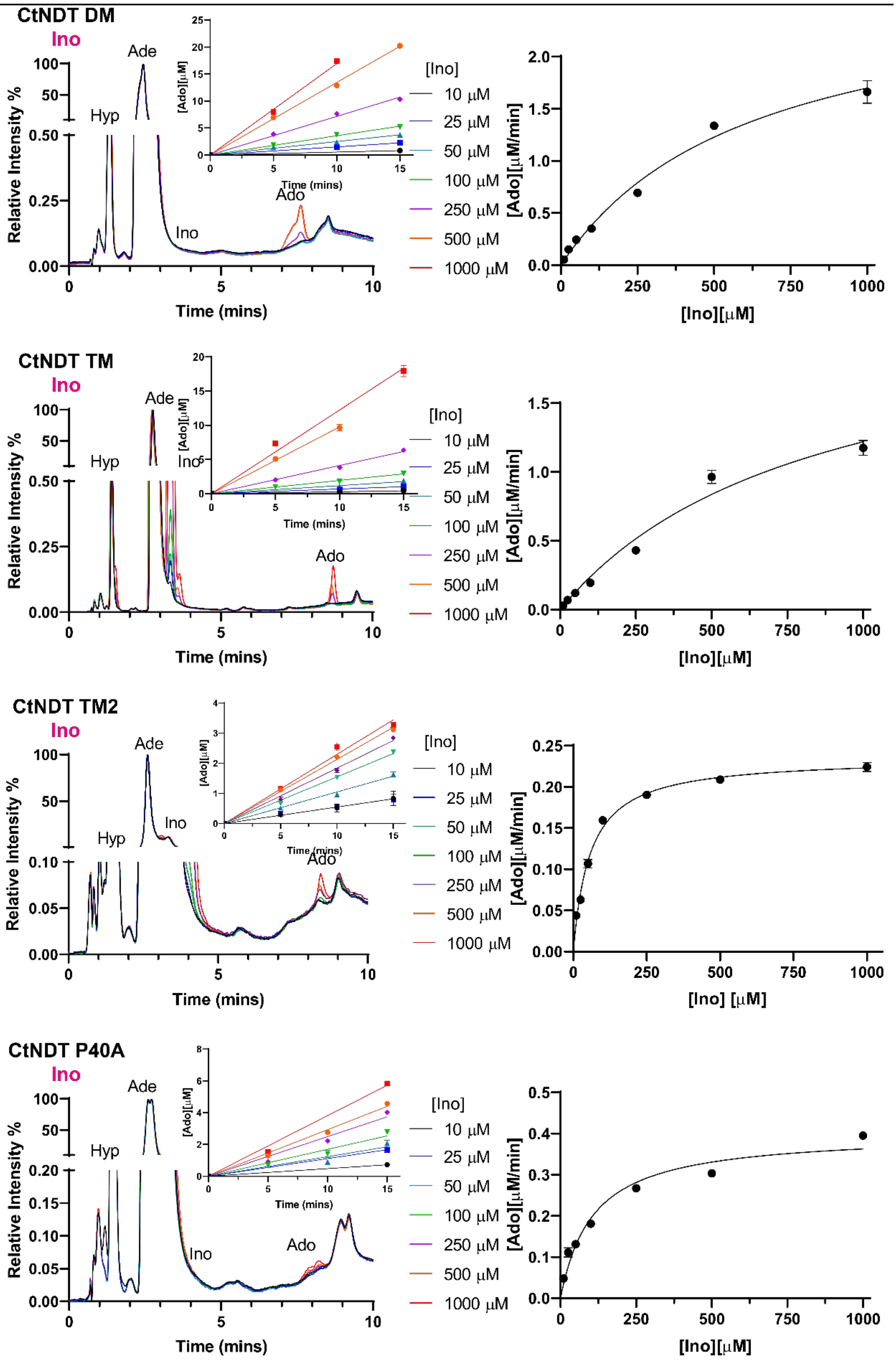

Figure S4. HPLC and raw kinetic data for all enzyme variants tested.

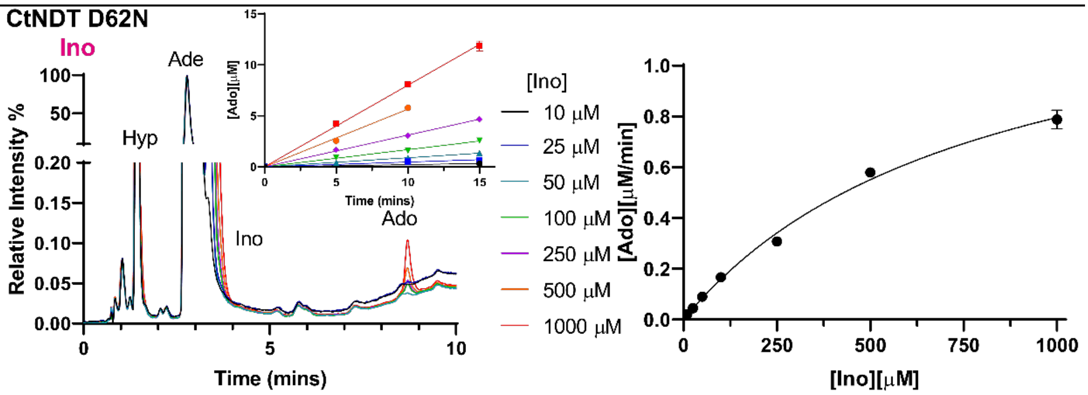

Figure S4. HPLC and raw kinetic data for all enzyme variants tested. Left, HPLC traces (260 nm absorbance), inset depicts replots of integrated area valus for each peak. A calibration curve with known quantities of Ado was used to convert are values into concentration. Right, replot of rates of product formation in function of substrate concentration to obtain kinetic parameters after fitting to a Michaelis-Menten equation.

Figure S5: Proof of concept for nucleoside production using CtNDT$_{Y7F\text{-}A9S}$. Our procedure generated nucleosides in a single step with the conditions shown below. Compound identities were confirmed by comparison to commercial standards of each new nucleoside product, and quantitified based on calibration curves with authentic standards.

a

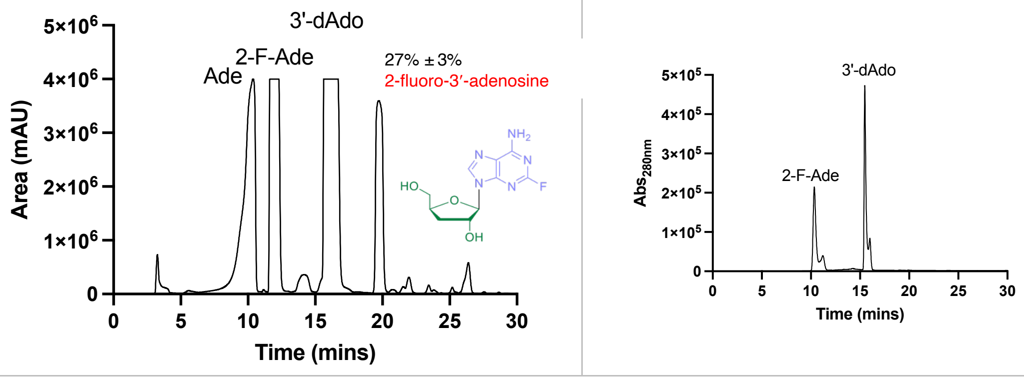

Left, HPLC trace (UV 260 nm, AU = arbitrary units) for purification of 2-fluoro-3′-deoxyadenosine (or 2F-3′d-Ado or 2F-cordycepin, Product 3’-deoxy-3). Reaction was carried out with 1:10 2-fluoroadenine to cordycepin substrate ratio. Quantifying 3′-deoxyadenosine decay reveals 27% of starting nucleoside substrate was hydrolysed. Right, reaction control with no enzyme added.

b

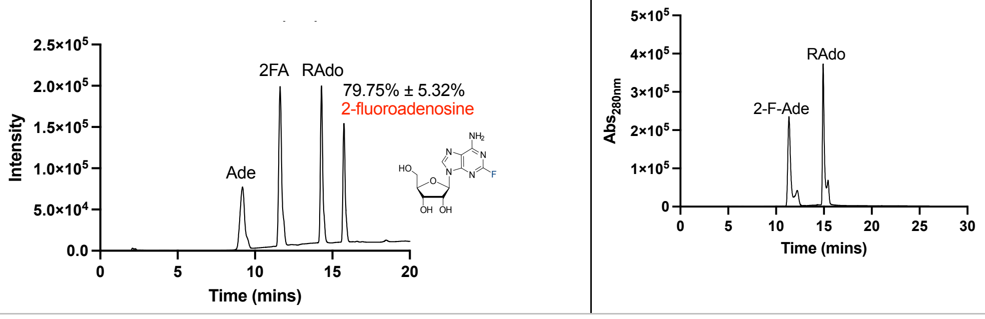

Left, HPLC trace (UV 260 nm)for purification of 2-fluoroadenosine (Product ribo-3). Quantifying adenosine decay reveals negligible hydrolysis. Right, reaction control with no enzyme added.

c

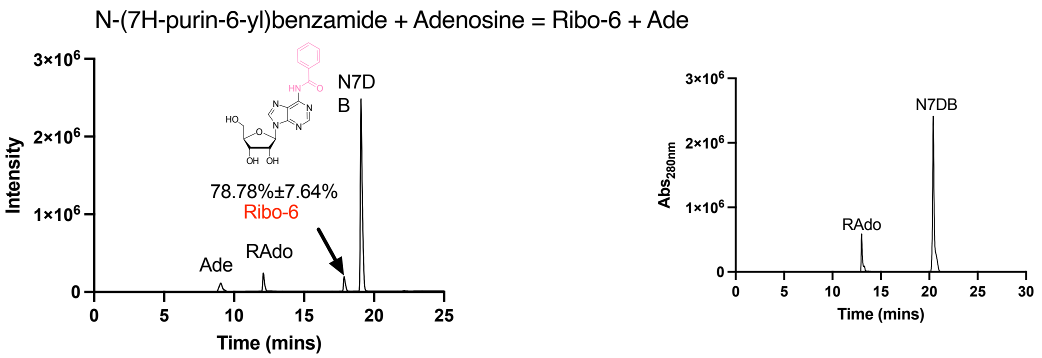

Left, HPLC trace (UV 260 nm) for purification of Product ribo-6. Reaction was carried out with 1:10 Adenosine to N-(9H-purin-6-yl)benzamide substrate ratio. Quantifying adenosine decay reveals negligible hydrolysis. Right, reaction control with no enzyme added.

d

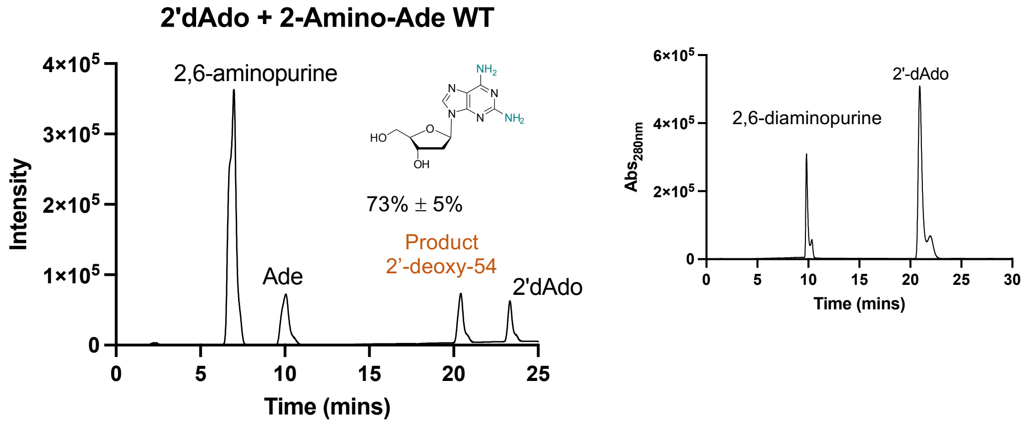

HPLC trace (UV 260 nm) for purification of Product 2'-deoxy-54 in a reaction with wild type enzyme. Reaction was carried out with 1:10 Adenosine to 2,6-diaminopurine substrate ratio. Quantifying 2′-deoxyadenosine decay reveals 15% hydrolysis. Right, reaction control with no enzyme added.

e

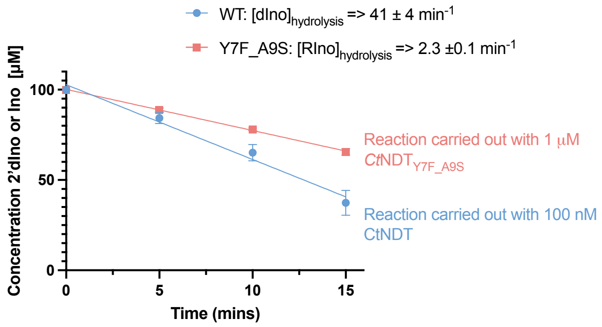

Time course for hydrolysis of 100 μM 2'-deoxyinosine using wild type *Ct*NDT or inosine using *Ct*NDT_{Y7F_A9S} in the presence of 1mM 2,6-diaminopurine.

Figure S6: Reaction tests to improve reaction yield and optimize nucleoside product formation. a) varying pH while using 1:1 2’-deoxy-guanosine and adenine as substartes, reaction conducted for different amounts of time as indicated on graph. b) varying temperature while using 1:1 2’-deoxy-guanosine and adenine as substrates. c) testing whether adding xanthine oxidase to reactions in which the nucleoside donor is inosive changes equilibrium. Reaction with 1:1 or 1:10 2'deoxyinosine and adenine as substrates.

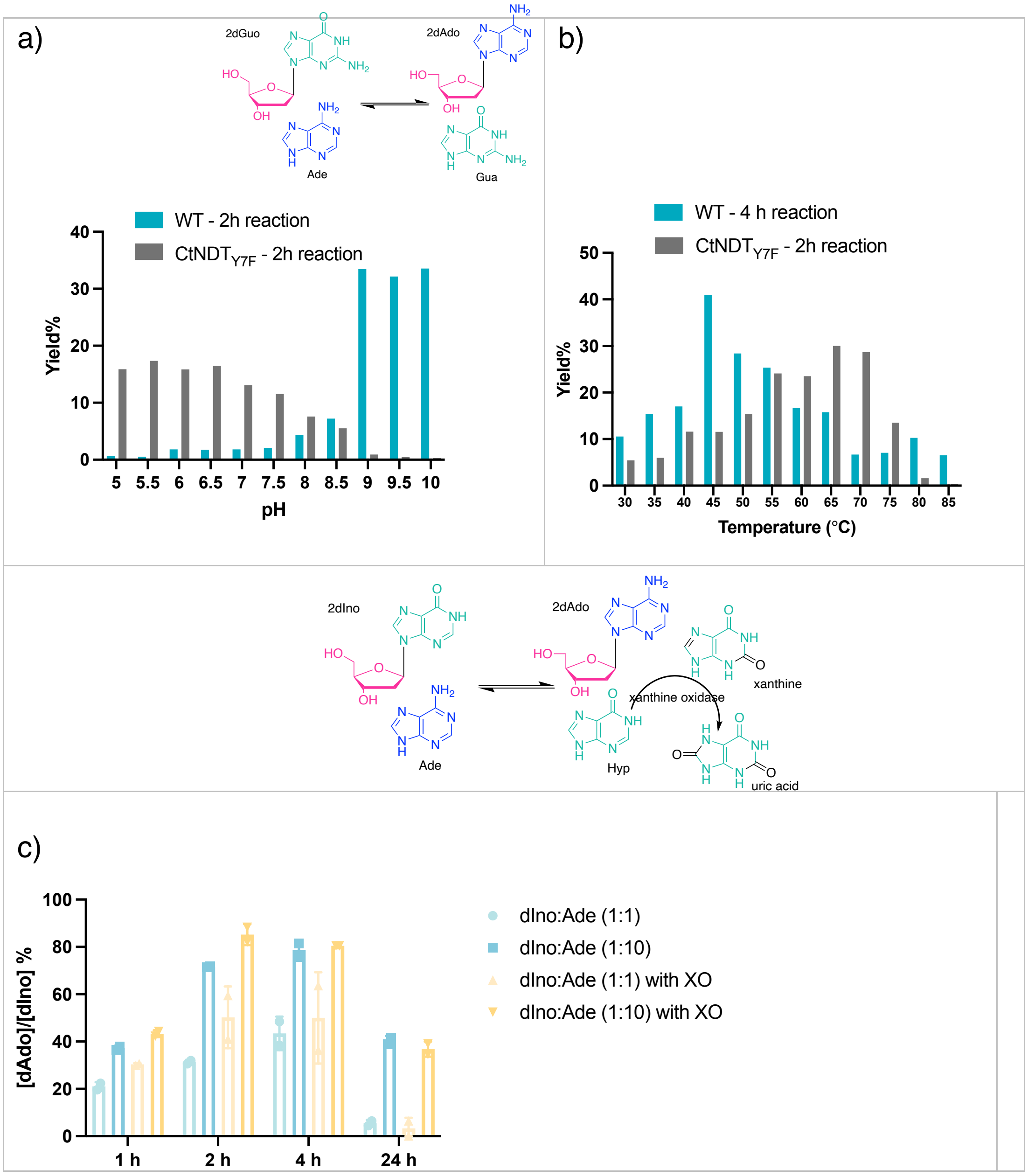

Figure S7: SDS-PAGE for all proteins used in this work. The orange boxes highlight fractions that were pooled and concentrated for use in subsequent experiments.

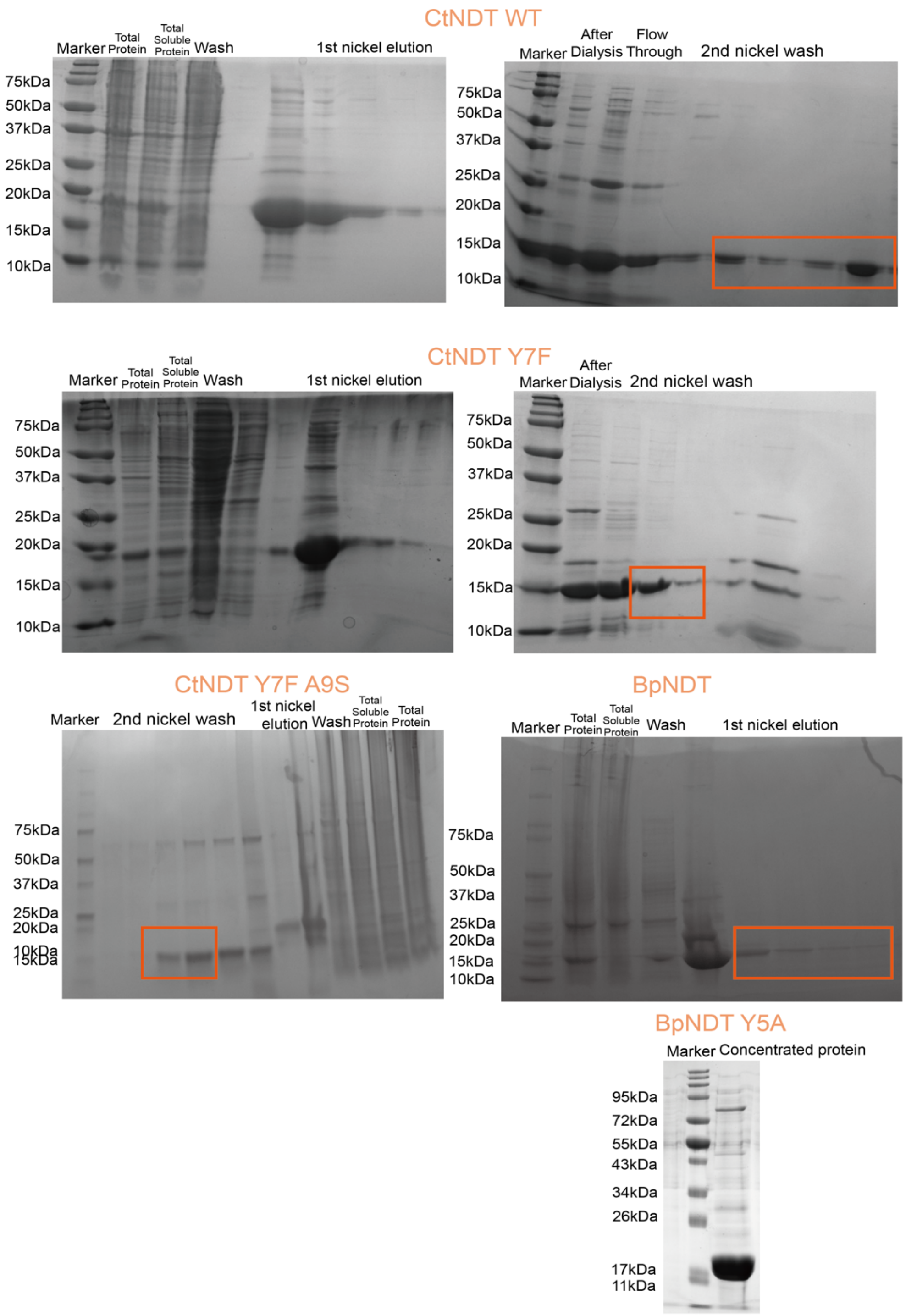

Figure S8: Intact protein mass spectrometry for all proteins employed in this work.

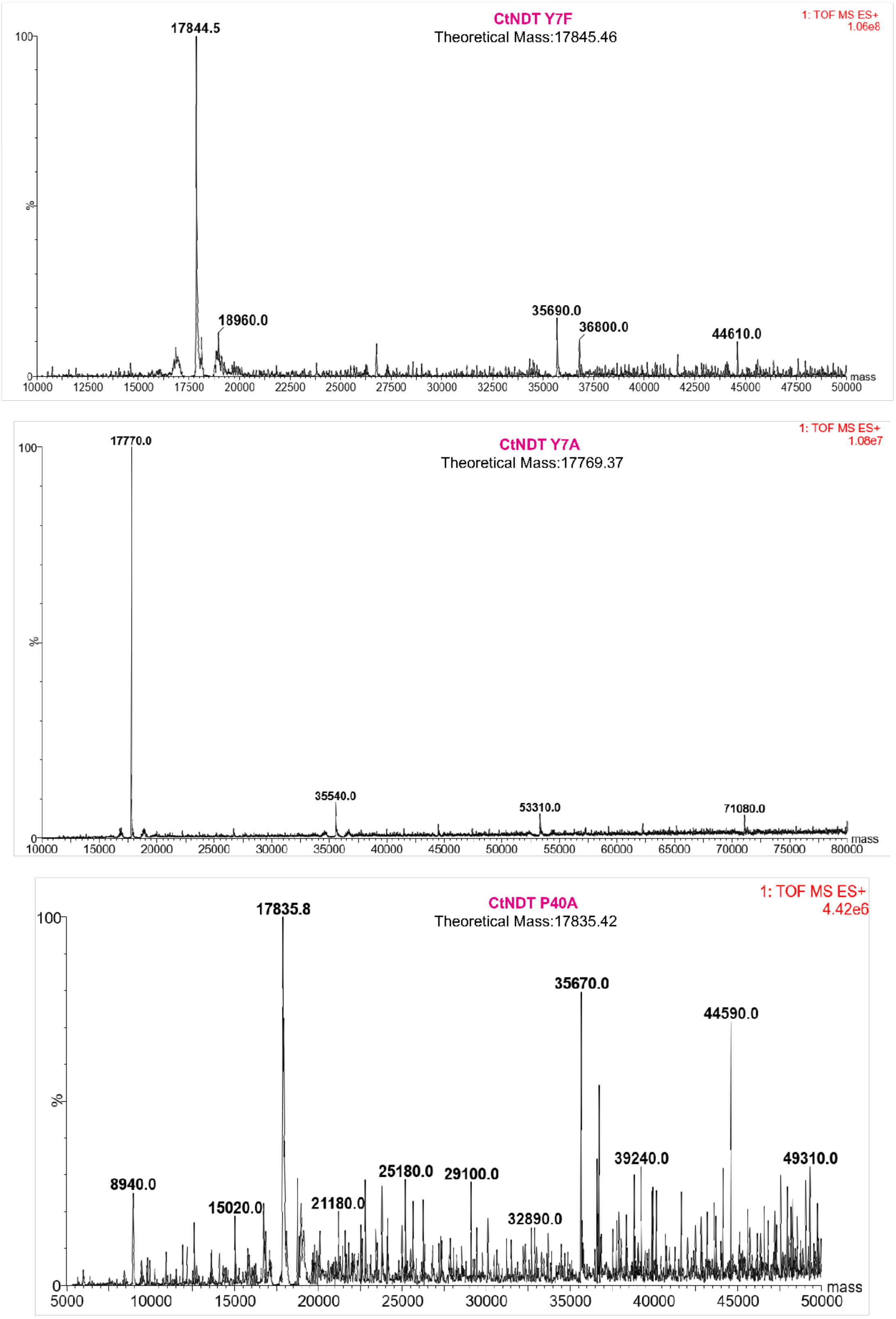

Figure S8: Intact protein mass spectrometry for all proteins employed in this work.

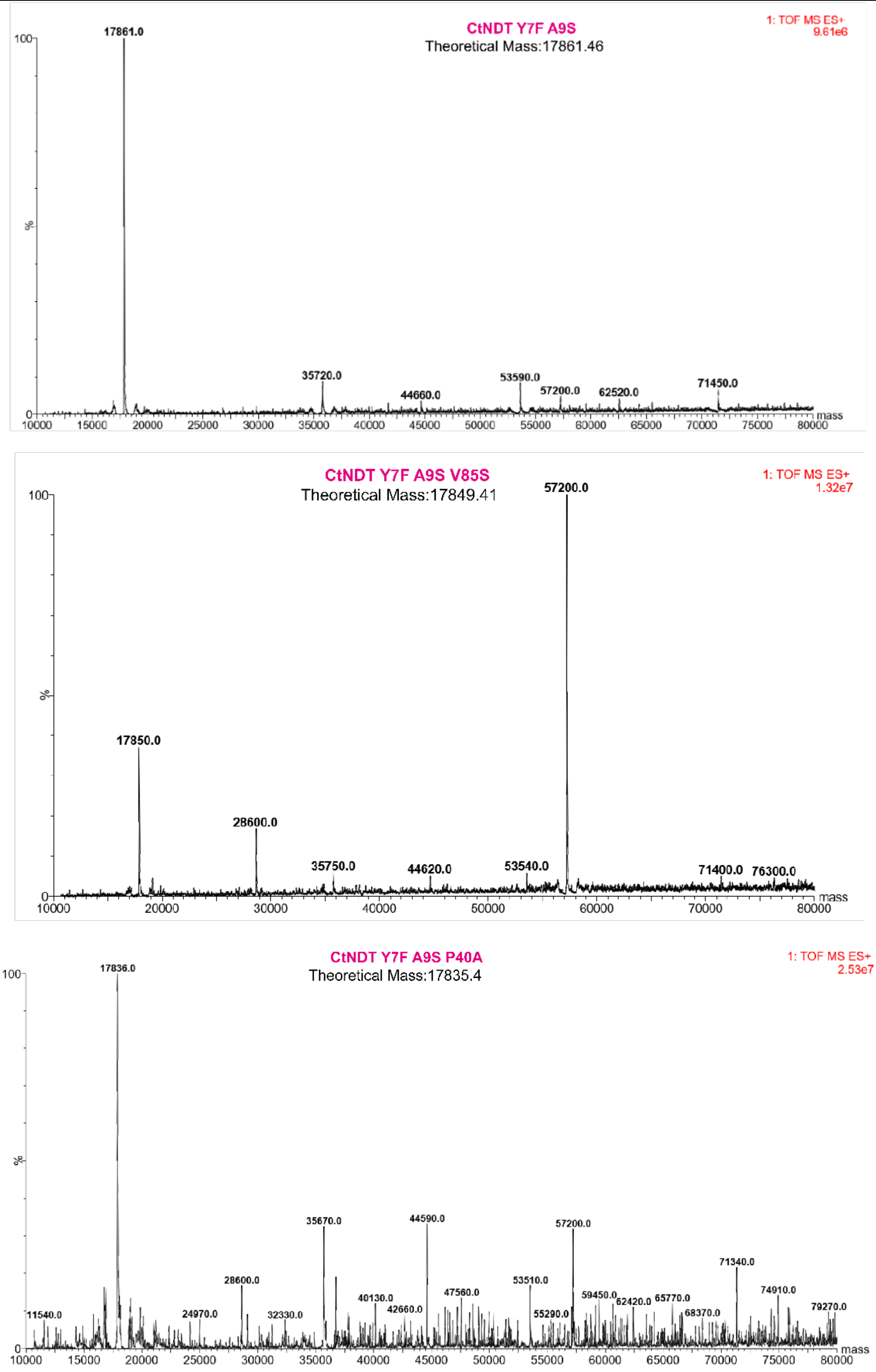

Figure S8: Intact protein mass spectrometry for all proteins employed in this work.

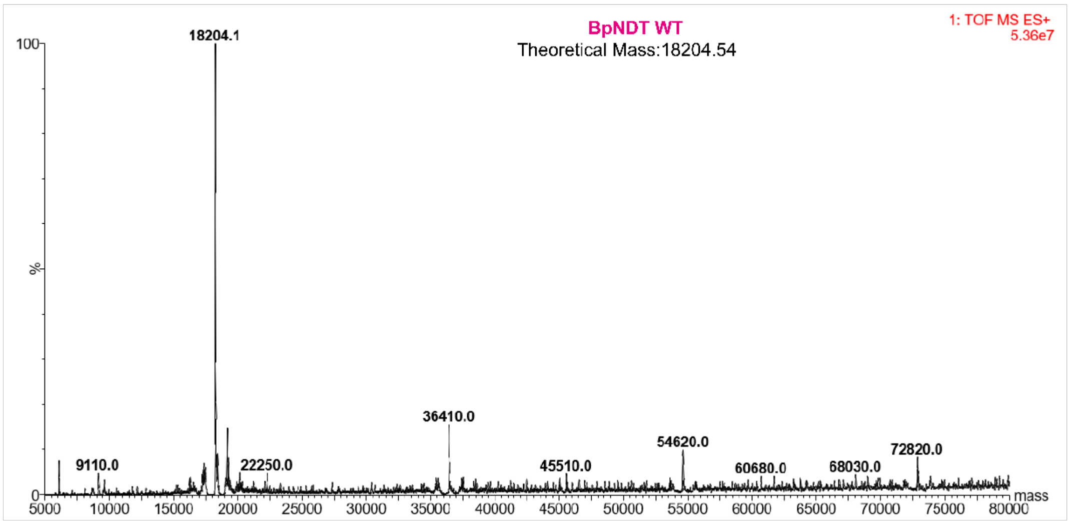

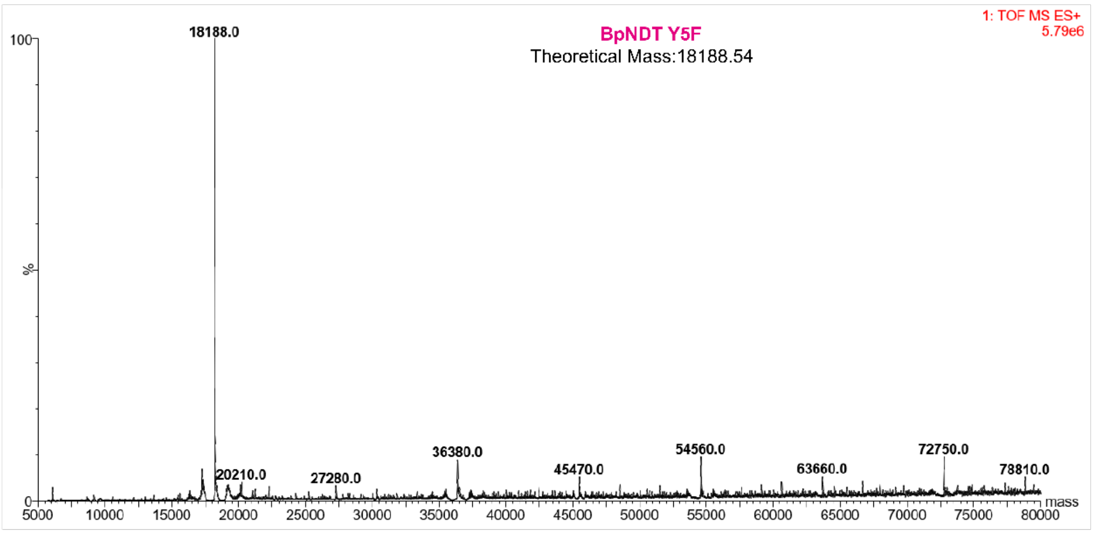

Figure S8: Intact protein mass spectrometry for all proteins employed in this work. Theoretical expected masses are shown as well as raw data.

Supporting References

1. Tang, P.; Harding, C. J.; Dickson, A. L.; da Silva, R. G.; Harrison, D. J.; Czekster, C. M., Snapshots of the Reaction Coordinate of a Thermophilic 2'-Deoxyribonucleoside/ribonucleoside Transferase. *ACS Catal* **2024,** *14* (5), 3090-3102.
2. Carberry, A. E.; Devi, S.; Harrison, D. J.; da Silva, R. G., Human 2'-Deoxynucleoside 5'-Phosphate N-Hydrolase 1: The Catalytic Roles of Tyr24 and Asp80. *Chembiochem* **2024**, e202400047.
3. Oke, M.; Carter, L. G.; Johnson, K. A.; Liu, H.; McMahon, S. A.; Yan, X.; Kerou, M.; Weikart, N. D.; Kadi, N.; Sheikh, M. A.; Schmelz, S.; Dorward, M.; Zawadzki, M.; Cozens, C.; Falconer, H.; Powers, H.; Overton, I. M.; van Niekerk, C. A.; Peng, X.; Patel, P.; Garrett, R. A.; Prangishvili, D.; Botting, C. H.; Coote, P. J.; Dryden, D. T.; Barton, G. J.; Schwarz-Linek, U.; Challis, G. L.; Taylor, G. L.; White, M. F.; Naismith, J. H., The Scottish Structural Proteomics Facility: targets, methods and outputs. *J Struct Funct Genomics* **2010,** *11* (2), 167-80.
4. Gasteiger, E.; Hoogland, C.; Gattiker, A.; Duvaud, S. e.; Wilkins, M. R.; Appel, R. D.; Bairoch, A., Protein Identification and Analysis Tools on the ExPASy Server. In *The Proteomics Protocols Handbook*, Walker, J. M., Ed. Humana Press: Totowa, NJ, 2005; pp 571-607.
5. Bai, N.; Roder, H.; Dickson, A.; Karanicolas, J., Isothermal Analysis of ThermoFluor Data can readily provide Quantitative Binding Affinities. *Sci Rep* **2019,** *9* (1), 2650.
6. Winn, M. D.; Ballard, C. C.; Cowtan, K. D.; Dodson, E. J.; Emsley, P.; Evans, P. R.; Keegan, R. M.; Krissinel, E. B.; Leslie, A. G.; McCoy, A.; McNicholas, S. J.; Murshudov, G. N.; Pannu, N. S.; Potterton, E. A.; Powell, H. R.; Read, R. J.; Vagin, A.; Wilson, K. S., Overview of the CCP4 suite and current developments. *Acta Crystallogr D Biol Crystallogr* **2011,** *67* (Pt 4), 235-42.
7. Schneider, B.; Gelly, J. C.; de Brevern, A. G.; Cerny, J., Local dynamics of proteins and DNA evaluated from crystallographic B factors. *Acta Crystallogr D Biol Crystallogr* **2014,** *70* (Pt 9), 2413-9.
8. Anand, R.; Kaminski, P. A.; Ealick, S. E., Structures of purine 2'-deoxyribosyltransferase, substrate complexes, and the ribosylated enzyme intermediate at 2.0 A resolution. *Biochemistry* **2004,** *43* (9), 2384-93.
